# Multisite Chronic Pain Reveals Neuro–Immune–Metabolic Dysregulation across Rheumatoid Arthritis and Depression

**DOI:** 10.34133/research.1298

**Published:** 2026-06-17

**Authors:** Qian Wang, Ye Ella Tian, Andrew Zalesky, Peng Wang, Zening Fu, Guozheng Feng, Dongmei Zhi, Ming Xu, Chunyang Wang, Xiaoli Wang, Xizhen Wang, Peiwu Qin, Vince D. Calhoun, Rongtao Jiang, Jing Sui

**Affiliations:** ^1^ State Key Laboratory of Cognitive Neuroscience and Learning, Beijing Normal University, Beijing, China.; ^2^ Department of Psychiatry, Melbourne Medical School, The University of Melbourne, Melbourne, Australia.; ^3^ Department of Biomedical Engineering, Faculty of Engineering and Information Technology, The University of Melbourne, Melbourne, Australia.; ^4^ Tri-institutional Center for Translational Research in Neuroimaging and Data Science (TReNDS), Georgia State University, Georgia Institute of Technology, and Emory University, Atlanta, GA 30303, USA.; ^5^ Center for Addiction Medicine, Massachusetts General Hospital, Boston, MA 02114, USA.; ^6^ Department of Psychiatry, Massachusetts General Hospital and Harvard Medical School, Boston, MA 02115, USA.; ^7^ China Mobile Research Institute, Beijing 100032, China.; ^8^ School of Education, Ludong University, Yantai 264025, China.; ^9^ School of Medical Imaging, Shandong Second Medical University, Weifang 261053, China.; ^10^ Medical Imaging Center Affiliated Hospital of Shandong Second Medical University, Weifang 261031, China.; ^11^ Hengqin Lab, Zhuhai, Guangdong, China.; ^12^ IDG/McGovern Institute for Brain Research, Beijing Normal University, Beijing, China.

## Abstract

Multisite chronic pain (MCP) frequently co-occurs with immune and depressive disorders, yet whether it reflects coordinated cross-domain multi-omic dysregulation remains unknown. Using UK Biobank data (19,484 baseline participants; 32,870 to 399,476 for 15.9-year follow-up), we identified MCP-related multi-omic signatures spanning 59 biochemical measures, 168 metabolites, and 2,920 proteins. Notably, these signatures showed graded dysregulation [controls < depression < rheumatoid arthritis (RA) < comorbidity] with increasing disease burden, were associated with increased risk of incident RA and depression, and partially mediated their bidirectional association. We further identified HNMT as a depression risk factor, FGF21 as an RA risk factor, MME as a depression protective factor, and platelet count/FGF21/HNMT as shared factors using Mendelian randomization. Beyond disease outcomes, the signatures were associated with brain structural impairment and health-related behaviors (smoking/fish intake/physical activity), and aligned with polygenic liability for immune–metabolic–psychiatric traits. Together, these findings demonstrate that MCP reflects coordinated neuro–immune–metabolic dysregulation underlying RA–depression comorbidity and functions as a systems-level phenotype linking immune processes and depression.

## Introduction

Rheumatoid arthritis (RA) is a chronic autoimmune disease characterized by systemic inflammation, joint damage, and substantial disability [1,2]. In 2021, approximately 17.9 million people worldwide were living with RA, and this number is projected to increase to 31.7 million by 2050 [3]. People with RA face a significant psychiatric burden, particularly depression [4,5]*.* Comorbid depression and RA are linked to intensified pain [6], poorer treatment adherence, and increased risks of long-term disability and mortality [7]. However, previous studies have often treated RA and depression as distinct conditions, limiting understanding of their shared biological mechanisms [7,8]. Elucidating these shared mechanisms is essential for developing effective therapeutic strategies to manage immune–depression comorbidities.

Clinically, chronic pain frequently co-occurs with RA and depression, and patients with RA consistently identify pain as their most important and burdensome symptom [6,9,10]*.* RA-related pain is often not confined to inflamed joints but instead manifests as multisite chronic pain (MCP), a widespread pain phenotype linked to heightened vulnerability to depression [11,12]. Recent genetic evidence demonstrates substantial correlations between MCP and RA [13], and our previous work has identified a potential causal effect of MCP on the development of depression [14], positioning MCP as a clinically meaningful and biologically informative nexus for understanding shared mechanisms underlying RA and depression.

Growing evidence indicates that RA, depression, and MCP share overlapping biological pathways involving immune cell-mediated inflammation [6,15,16], disrupted biochemical profiles [9], and metabolic disturbances [17–20], often reflected in peripheral blood biomarkers. However, existing research has largely examined isolated molecular markers, making it difficult to integrate the complex, multi-system processes underlying RA, depression, and chronic pain [8]. Multi-omic integration enables biochemical, metabolic, and proteomic signals to be examined jointly, providing a broader view of coordinated perturbations in immune and metabolic pathways [21,22]. Despite this potential, systematic cross-omic analyses that characterize the shared biological architecture linking MCP, RA, and depression remain scarce. Importantly, peripheral inflammatory and metabolic disturbances may also influence the brain through blood–brain barrier-mediated signaling [23,24]. Consistent with this, neuroimaging studies show that the anterior cingulate cortex and prefrontal cortex, key regions for pain–emotion integration, exhibit heightened functional activity in RA and depression [6,7,25]. Yet, it remains unclear how the multi-omic architecture underlying MCP is reflected in brain-wide structural organization or how these signatures relate to polygenic susceptibility across physical and psychiatric phenotypes. Clinically, pain in RA often persists despite optimal inflammatory control [26,27], highlighting the need to identify MCP-related phenotypic factors that can inform more effective pain-management strategies [28].

To address these gaps, we conceptualized MCP as a quantifiable and clinically accessible systems-level phenotype linking RA and depression. Leveraging peripheral multi-omic data from the UK Biobank, we applied MCP-guided multi-omic integration [29–31] across biochemical, metabolomic, and proteomic layers to derive MCP-associated molecular signatures in controls, depression, RA, and their comorbidity. Specifically, we aimed to (a) delineate peripheral multi-omic signatures of MCP and examine whether these signatures exhibit graded dysregulation with increasing disease burden, (b) evaluate the prospective associations between these identified signatures and the subsequent onset of RA and depression, (c) assess their mediating role in the bidirectional association between RA and depression, (d) identify potential causal risk and protective factors for depression and RA, and (e) characterize how these signatures relate to polygenic susceptibility, brain structural metrics, and 674 health-related phenotypes. This framework provides a systems-level perspective on multisite pain as a biological bridge between immune processes and depression.

## Results

We leveraged peripheral multi-omic data from the UK Biobank, including 59 blood cell count and biochemistry measures, 168 metabolites quantified using the Nightingale Health proton nuclear magnetic resonance (NMR) spectroscopy platform, and 2,920 proteins. The MCP score, derived from a touchscreen questionnaire assessing the number of body sites with pain lasting longer than 3 months (range, 0 to 7), was used as a continuous phenotype to guide a supervised multi-omic integration framework in healthy controls (participants without mental health disorders or immune-related arthritis at baseline, *n* = 5,605, mean age = 56.23 years, 45% female; see Table S15 for full characteristics).

**Fig. 1.**
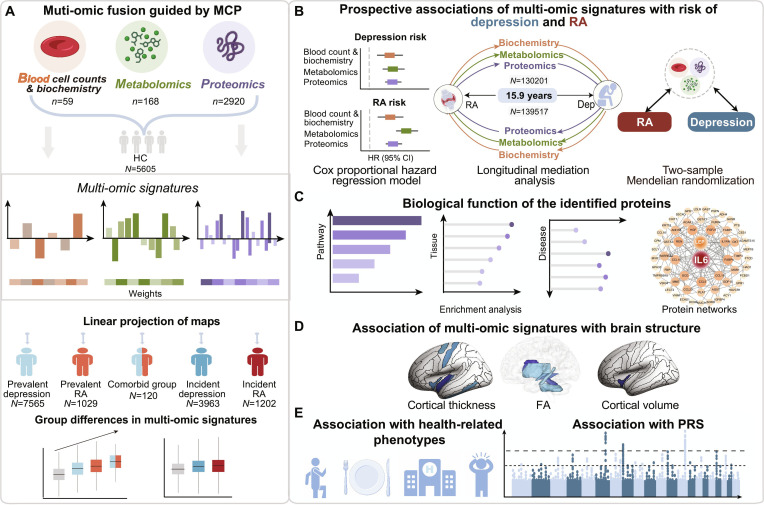
Study overview. (A) Multisite chronic pain (MCP) scores were used as the reference to guide a multi-omic fusion of data comprising blood cell counts/biochemistry, metabolites, and proteomic profiles from healthy controls (HC) by supervised learning. The derived MCP-related multi-omic signatures were subsequently back-reconstructed in 5 patient groups (prevalent depression, prevalent RA, RA–depression comorbidity, incident depression, and incident RA) to assess the group-level deviations relative to HCs. (B) Prospective associations of the identified signatures with depression and RA, their mediating effects between RA and depression bidirectionally, and their causal effect inference on depression and RA by MR. (C) Biological function evaluation of the identified proteins through tissue expression profiling, enrichment analyses of KEGG pathways, GO biological processes, disease-association analyses, and PPI network analysis. (D) Associations with brain structural measures, including CT, white-matter FA, and CV. (E) Associated with PRSs and health-related phenotypes. Some icons were created with BioRender.com.

The MCP-associated multi-omic signatures identified in healthy controls were subsequently projected to 5 patient groups defined by International Classification of Diseases (ICD)-coded diagnoses (Materials and Methods), including prevalent depression (depression diagnosed at or before the baseline assessment, *n* = 7,565), prevalent RA (RA diagnosed at or before the baseline assessment; *n* = 1,029), RA–depression comorbidity (diagnosed with both depression and RA at or before the baseline assessment, *n* = 120), incident depression (new-onset cases during follow-up, *n* = 3,963), and incident RA (new-onset cases during follow-up, *n* = 1,202) to determine whether these signatures exhibit graded changes consistent with increasing disease burden (Materials and Methods). Descriptive distributions of MCP scores across study cohorts are provided in Table S16. Then, the identified multi-omic signatures were investigated further through prospective prediction, association analyses, and causal inference to define their involvement in RA and depression (Fig. 1). These analyses were designed to capture complementary aspects of the relationships between MCP, multi-omic signatures, and disease outcomes.

### MCP-related peripheral multi-omic signatures

**Fig. 2.**
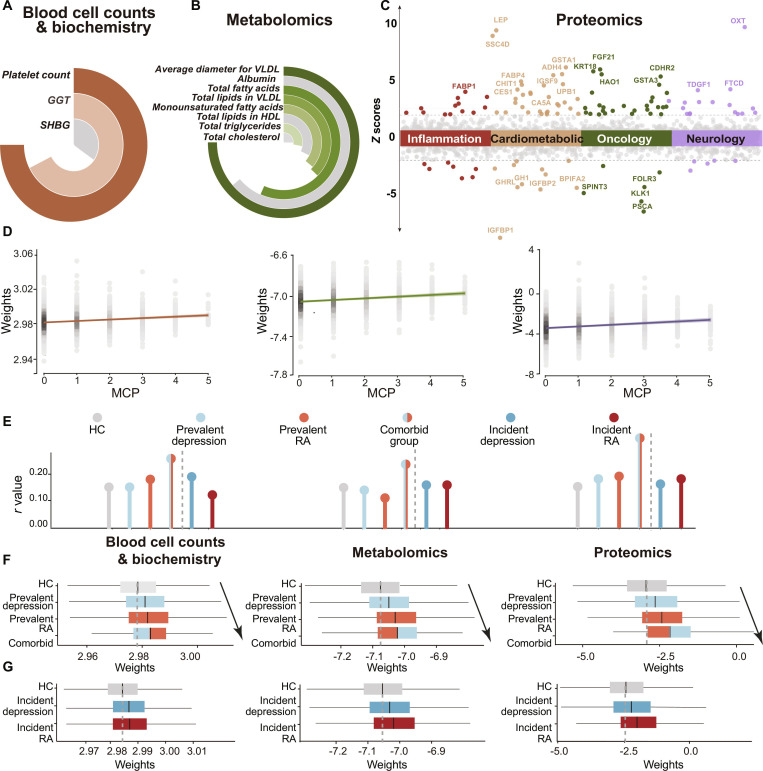
MCP-related multi-omic fusion results. (A) Three MCP-associated signatures in blood cell counts and biochemistry (|*Z*| > 2); segment length indicates |*Z*|, with red and gray denoting positive and negative associations. (B) Eight MCP-associated signatures in metabolomics (|*Z*| > 2); green and gray denote positive and negative associations. (C) Manhattan plot of 2,920 plasma proteins showing 119 MCP-associated proteomic signatures; nonsignificant proteins (|*Z*| < 2) are shown in gray. (D) Correlations between MCP scores and weights derived from blood cell counts/biochemistry, metabolomics, and proteomics in the HC group, respectively. Higher weights were associated with higher MCP scores, indicating a greater multisite pain burden. (E) Significant correlations between MCP scores and weights across all omics were also observed across groups. (F) One-way ANOVA with polynomial trend analysis showing a linear increase in deviation from HCs across prevalent depression, prevalent RA, and comorbid RA–depression; values were linearly scaled for visualization. (G) Significant differences were also observed between incident RA/incident depression group and HCs. Boxplots show median, IQR, and 1.5× IQR.

Among healthy UK Biobank participants (*N* = 5,605; see Fig. S1 for participant selection in the fusion analyses), our supervised multi-omic integration framework revealed MCP-related signatures across blood cell counts/biochemistry, metabolomics, and proteomics (Fig. 2A to C). After regressing out covariates and controlling the false discovery rate (FDR) (Materials and Methods), multi-omic signatures weights were positively correlated with MCP in blood cell counts/biochemistry (*r* = 0.16, *P* = 2.24 × 10^−33^), metabolomics (*r* = 0.16, *P* = 1.67 × 10^−33^), and proteomics (*r* = 0.16, *P* = 1.69 × 10^−32^) (Fig. 2D), indicating that higher molecular weights corresponded to greater multisite pain burden. Specifically, key MCP-related signatures in the blood cell counts and biochemistry domain included γ-glutamyltransferase (GGT), sex hormone-binding globulin (SHBG), and platelet count (Fig. 2A). Metabolomic signatures associated with MCP comprised 8 features: total cholesterol, total triglycerides, total lipids in very low-density lipoproteins (VLDLs) and high-density lipoproteins (HDLs), average VLDL particle diameter, total fatty acids, monounsaturated fatty acids, and albumin (Fig. 2B). Proteomic signatures related to MCP comprised 119 plasma proteins, such as FGF21, LEP, NMNAT, IGFBP1, and GHRL (Fig. 2C). Repeating the fusion analyses in healthy controls using more stringent inclusion criteria yielded consistent results (Fig. S2).

To further assess the disease relevance of the identified multi-omic signatures, we projected these signatures across groups with varying disease status. This analysis revealed consistent associations across patient groups, including prevalent depression (blood biochemistry: *r* = 0.16, *P =* 9.80 × 10^−46^; metabolomics: *r* = 0.15, *P =* 3.02 × 10^−37^; proteomics: *r* = 0.19, *P =* 1.88 × 10^−25^; Fig. 2E and Fig. S3B), prevalent RA (blood biochemistry: *r* = 0.19, proteomics: *P* = 9.45 × 10^−10^; metabolomics: *r* = 0.12, *P =* 1.06 × 10^−4^; proteomics: *r* = 0.2, *P =* 3.11 × 10^−5^; Fig. 2E and Fig. S3A), RA–depression comorbidity (blood biochemistry: *r* = 0.27, *P =* 2.47 × 10^−3^; metabolomics: *r* = 0.25, *P* = 6.70 × 10^−3^; proteomics: *r* = 0.35, *P =* 1.21 × 10^−2^; Fig. 2E and Fig. S3C), incident depression (blood biochemistry: *r* = 0.2, *P =* 2.23 × 10^−37^; metabolomics: *r* = 0.17, *P =* 1.08 × 10^−26^; proteomics: *r* = 0.17, *P =* 1.40 × 10^−11^; Fig. 2E and Fig. S3E), and incident RA (blood biochemistry: *r* = 0.13, *P =* 1.19 × 10^−5^; metabolomics: *r* = 0.17, *P =* 6.61 × 10^−9^; proteomics: *r* = 0.19, *P =* 2.02 × 10^−5^; Fig. 2E and Fig. S3D).

### Linear gradients of MCP-related multi-omic signatures across disease burden

To further examine whether the identified multi-omic signatures exhibit a graded pattern across increasing disease burden, we performed one-way analysis of variance (ANOVA) followed by polynomial trend analysis. As shown in Fig. 2F, the identified multi-omic signatures showed significant differences across disease burden, with projection weights derived from blood cell counts and biochemistry displaying a significant overall group difference [*F* (3, 14,315) = 76.84, *P* < 2 × 10^−16^] and a robust linear trend (*t* = 4.73, *P* = 2.26 × 10^−6^) across disease burden. Consistent with this pattern, case–control effects increased from depression (Cohen’s *d* = 0.24, *P* = 9.8 × 10^−46^) to RA (*d* = 0.35, *P* = 9.45 × 10^−10^) and were largest in the comorbid group, consistent with the overall gradient pattern (*d* = 0.43, *P* = 2.5 × 10^−3^). A similar linear gradient was observed in metabolomics [*F* (3, 14,315) = 125.5, *P* < 2 × 10^−16^; *t* = 6.95, *P* = 3.74 × 10^−12^], with effect sizes increasing from depression (*d* = 0.28, *P* = 3.02 × 10^−37^) to RA (*d* = 0.51, *P =* 1.06 × 10^−4^) and comorbidity (*d* = 0.59, *P* = 6.70 × 10^−3^). Proteomics showed the same graded progression [*F* (3, 9,166) = 88.37, *P* < 2 × 10^−16^; *t* = 6.47, *P* = 1.04 × 10^−10^], with effects increasing from depression (*d* = 0.30, *P* = 1.88 × 10^−25^) to RA (*d* = 0.49, *P* = 3.11 × 10^−5^) and showing the highest effect size in the comorbid group, consistent with the overall gradient pattern (*d* = 0.88, *P* = 0.01). Together, these findings indicated a graded pattern of deviation in MCP-related multi-omic signatures across disease burden. Sensitivity analyses based on 1,000 bootstrap iterations with matched sample sizes across groups revealed a consistent stepwise trend across 3 omic layers (Fig. S4). Consistent results were also observed in repeated resampling analyses, in which groups were matched to the size of the comorbidity group, and the linear trend remained significant in the vast majority of iterations (>96%) across all omic layers (Fig. S11).

We compared the identified multi-omic signatures between healthy controls and individuals who subsequently developed depression or RA to determine whether the signatures reflect early molecular alterations preceding clinical onset. Significant differences were evident for incident depression (blood biochemistry: *d* = 0.24, *P =* 2.23 × 10^−37^; metabolomics: *d* = 0.26, *P =* 1.08 × 10^−26^; proteomics: *d* = 0.33, *P =* 1.40 × 10^−11^; Fig. 2G) and for incident RA (blood biochemistry: *d* = 0.25, *P =* 1.19 × 10^−5^; metabolomics: *d* = 0.38, *P =* 6.61 × 10^−9^; proteomics: *d* = 0.40, *P =* 2.01 × 10^−5^; Fig. 2G). These consistent abnormalities remained robust in sensitivity analyses using 1,000 bootstrap iterations with group-matched sample sizes across all omic layers (Fig. S5), suggesting that peripheral omic perturbations may precede clinical onset.

### Prospective association between the identified signatures and incidence of depression and RA

**Fig. 3.**
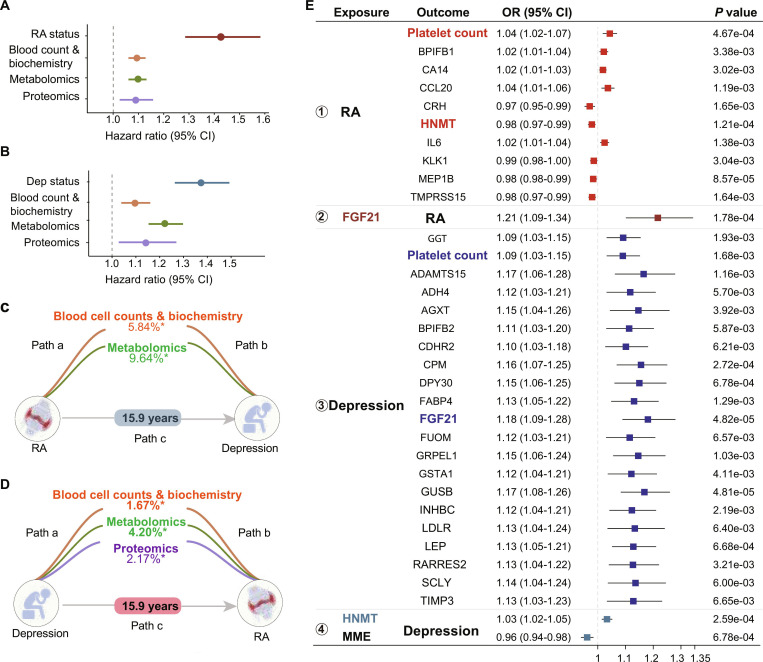
Prospective and causal associations of the identified multi-omic signatures with incident depression and RA. (A) Baseline RA status and the identified multi-omic signatures were associated with the risk of depression. (B) Baseline depression status and the identified multi-omic signatures were associated with the risk of RA. (C) Blood cell counts and biochemistry signatures, and metabolomic signatures, partially mediated the prospective association between baseline RA and incident depression. (D) Blood cell counts and biochemistry, metabolomic, and proteomic signatures partially mediated the prospective association between baseline depression and incident RA. (E) MR analyses revealed significant bidirectional causal relationships between the identified multi-omic signatures and the risk of depression and RA. The 3 color-highlighted proteins: Platelet count, FGF21, and HNMT were identified in both RA- and depression-related MR analyses. FDR correction (FDR < 0.05) was applied separately to each of the 4 categories of MR analysis.

Building on the cross-sectional associations, we next examined whether the identified multi-omic signatures were prospectively associated with incident depression and RA. We used data from 371,271 depression-free individuals with a median follow-up of 13.72 years [interquartile range (IQR): 12.99 to 14.42] to study the prospective association between baseline RA and depression incidence. Compared with individuals without RA, those with RA were associated with a 42% [hazard ratio (HR) = 1.42, 95% confidence interval (CI): 1.28 to 1.58, *P =* 2.55 × 10^−11^] higher risk of developing depression, after adjusting for covariates including age, sex, body mass index (BMI), alcohol consumption, smoking status, ethnicity, household income, education level, deprivation index, and metabolic syndrome (defined as the presence of ≥3 of central obesity, diabetes, hypertension, low HDL cholesterol, or elevated triglycerides) (Fig. 3A). Analyses based on 399,476 RA-free individuals showed that baseline depression was associated with a 38% (HR = 1.38, 95% CI: 1.27 to 1.50, *P =* 6.32 × 10^−14^) higher risk of developing RA over a median follow-up of 13.77 years (IQR: 13.05 to 14.44), after adjusting for the same set of covariates (Fig. 3B).

Significant associations were identified between the multi-omic signatures and the incidence of both depression and RA in a cohort of more than 32,870 participants. Specifically, these signatures were associated with an increased risk of depression (blood biochemistry: HR = 1.09, 95% CI: 1.06 to 1.13, *P* = 1.58 × 10^−8^; metabolomic: HR = 1.10, 95% CI: 1.06 to 1.13, *P* = 1.56 × 10^−8^; proteomic: HR = 1.09, 95% CI: 1.03 to 1.16, *P* = 4.01 × 10^−3^; Fig. 3A) and RA (blood biochemistry: HR = 1.10, 95% CI: 1.04 to 1.16, *P* = 9.48 × 10^−4^; metabolomic: HR = 1.22, 95% CI: 1.15 to 1.30, *P* = 1.52 × 10^−11^; proteomic: HR = 1.14, 95% CI: 1.03 to 1.27, *P* = 0.012; Fig. 3B). All associations remained significant after FDR correction (FDR < 0.05) and persisted when excluding individuals experiencing events during the first 2 years of follow-up (Fig. S7). In addition, we evaluated the discriminative performance of the identified multi-omic signatures using survival-based metrics. The C-index values ranged from 0.63 to 0.67 across different multi-omic modalities for both incident depression and incident RA, indicating modest but consistent discrimination (Table S17). Furthermore, individuals in the high-risk group had approximately twice the risk of those in the low-risk group for both incident depression (HRs, 2.01 to 2.16; *P* < 4.23 × 10^−48^) and incident RA (HRs, 1.97 to 2.67; *P* < 8.99 × 10^−24^; Table S18), indicating that these signatures effectively stratify risk.

Moreover, mediation analyses identified small but significant mediating effects of the identified multi-omic signatures on the RA–depression association. Specifically, blood cell counts and biochemistry signatures explained 5.84%, and metabolomic signatures explained 9.64%, of the association between RA and later depression (Fig. 3C). In the reverse direction, blood cell counts and biochemistry signatures explained 1.67%, metabolomic signatures 4.20%, and proteomic signatures 2.17% of the association (Fig. 3D).

### Causal associations of the identified multi-omic signatures with depression and RA

To further investigate potential causal relationships, we further conducted bidirectional Mendelian randomization (MR) analyses to evaluate the potential causal association of the identified multi-omic signatures (119 proteins, 8 metabolites, and 3 blood cell counts and biochemistry traits) with depression and RA. We used the inverse-variance weighted (IVW) method under a random-effects model as the primary method. We performed 4 categories of MR analyses, each assessing the association direction per disease, with FDR correction applied independently within each analysis category. We identified 34 significant causal associations, with the implicated signatures broadly mapping onto neurobiological, immunological, and metabolic functional categories (Fig. 3E). Protein-to-disease analyses revealed significant causal effects of HNMT and MME on depression [HNMT: odds ratio (OR) = 1.03, 95% CI: 1.02 to 1.05, *P* = 2.59 × 10^−4^; MME: OR = 0.96, 95% CI: 0.94 to 0.98, *P* = 6.78 × 10^−4^]. We also observed a deleterious causal effect of FGF21 on RA risk (OR = 1.21, 95% CI: 1.09 to 1.34, *P* = 1.78 × 10^−4^), positioning FGF21 as a risk factor for RA. In the reverse direction, a total of 10 and 21 peripheral signatures were identified as potential downstream consequences of RA and depression, respectively. Notably, platelet count, FGF21, and HNMT emerged as overlapping causal factors in both RA and depression, suggesting shared causal molecular pathways linking the 2 conditions. For associations showing evidence of horizontal pleiotropy, MR pleiotropy residual sum and outlier (MR-PRESSO) was used to remove outlier variants, which did not materially alter the results. Sensitivity analyses using the weighted median, simple median, and MR-Egger methods yielded consistent estimates (Tables S8 and S9). Leave-one-single-nucleotide polymorphism (SNP)-out analyses confirmed that no SNP drove the overall results (Fig. S8).

### Functional enrichment and protein–protein interaction analysis

**Fig. 4.**
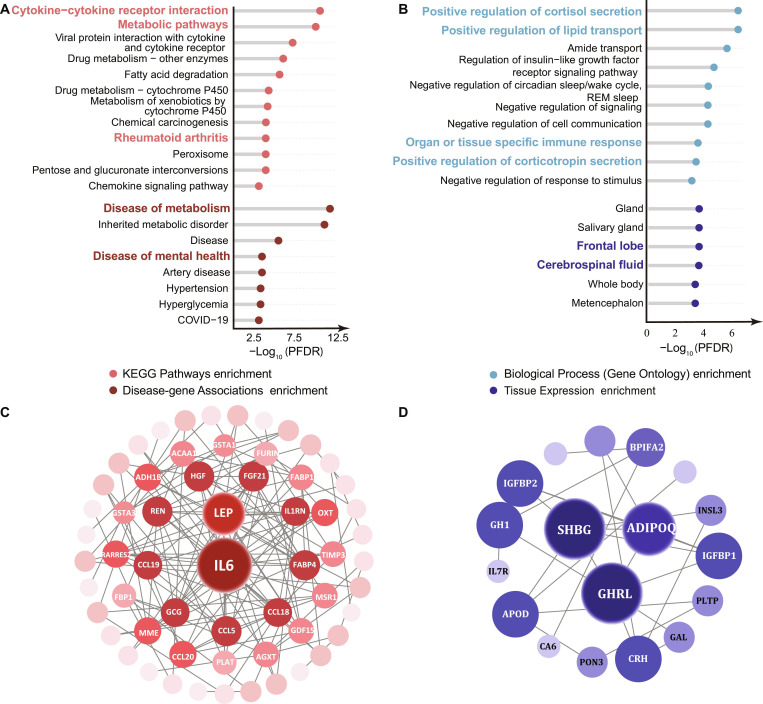
Functional analysis of MCP-associated proteins. (A) The MCP positively associated proteins were significantly enriched in KEGG pathways and disease–gene associations. The *x* axis showed log-transformed FDR-adjusted *P* values, and only terms that remained statistically significant after correction (FDR < 0.05) were displayed. The *y* axis listed the enriched terms, with colors indicating annotation sources. (B) The MCP negatively associated proteins were significantly enriched in GO categories, including Biological Process, Cellular Component, and Molecular Function, as well as tissue-specific expression profiles. (C) PPI network of MCP positively associated proteins. Each node represented a protein, with darker colors indicating higher degrees of connectivity. (D) PPI network of MCP negatively associated proteins, with node shading similarly reflecting connectivity degree.

Enrichment and protein–protein interaction (PPI) network analyses of the identified proteins (*N* = 119) were conducted to elucidate biological mechanisms potentially linking RA and depression. Proteins positively associated with MCP were enriched in immune- and RA-related pathways and in gene associations linked to mental health and metabolic disorders (Fig. 4A). In parallel, proteins negatively associated with MCP showed enrichment in brain and cerebrospinal tissues, and in biological processes related to immune responses, metabolic regulation, and cortisol and corticotropin secretion, suggesting involvement of hypothalamic–pituitary–adrenal (HPA) axis-related neuroendocrine pathways (Fig. 4B). These patterns converge on interconnected immune, neurobiological, and metabolic processes that may contribute to the biological basis of the comorbidity between depression and RA.

PPI analysis of MCP-related proteins revealed IL6 and LEP as hubs in the positive association network (Fig. 4C), while SHBG, GHRL, and ADIPOQ act as central nodes in negative association network (Fig. 4D).

### Association between the identified multi-omic signatures and brain structure

**Fig. 5.**
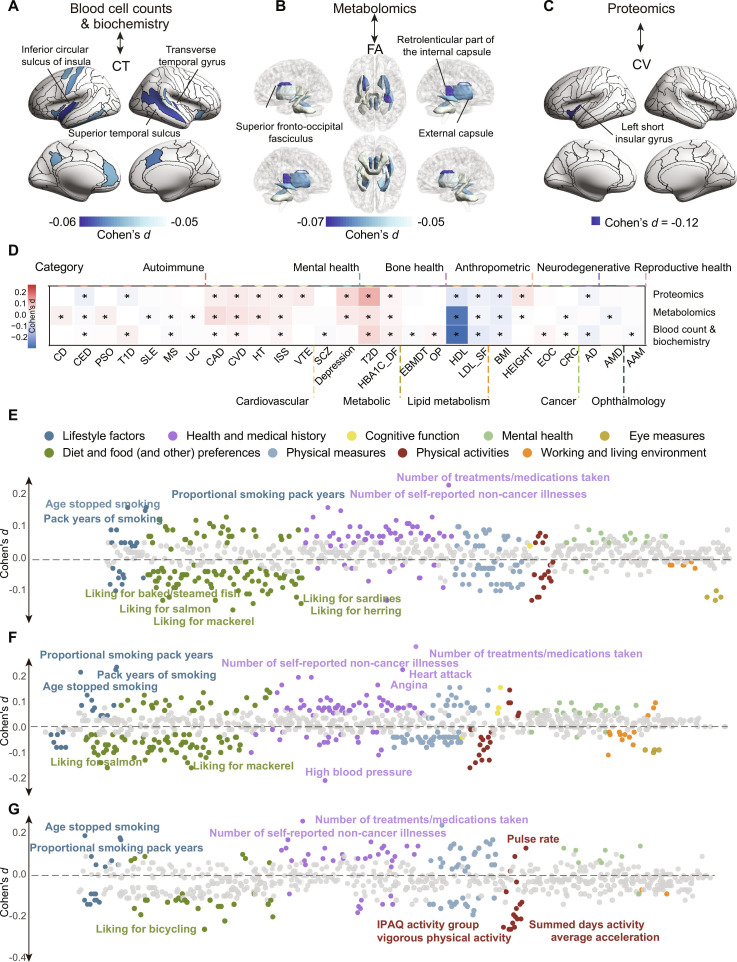
Associations of multi-omic signatures with brain structure, PRSs, and health-related phenotypes. (A) Associations of blood cell count and biochemistry signatures with CT; labeled regions show the 3 largest effects (Cohen’s *d*). (B) Associations of metabolomic signatures with FA. (C) Associations of proteomic signatures with CV; only the left short insular gyrus reached significance. All brain associations were estimated using linear mixed-effects models and passed FDR correction. (D) Associations with PRSs for diseases and physical traits; FDR-significant results (FDR < 0.05) were marked by asterisks. (E) Of 674 health-related phenotypes, 278 were associated with blood cell count and biochemistry signatures at a Bonferroni threshold (*P* < 7.42 × 10^−5^) after covariate adjustment. (F) The 299 phenotypes were associated with metabolomic signatures at the same threshold. (G) The 150 phenotypes were associated with proteomic signatures; nonsignificant phenotypes were shown in gray (top 10 labeled).

To determine how MCP-related multi-omic signatures were reflected in brain structure, we examined their associations with structural brain measures while controlling for the covariates used in the Cox and mediation models. We included intracranial volume as an additional covariate and imaging site as a random effect. The identified signatures were associated with widespread reductions in cortical thickness (CT), cortical volume (CV), and white-matter fractional anisotropy (FA). All associations remained significant after FDR correction (FDR < 0.05). Specifically, in the blood cell count and biochemistry domains, the identified signatures were predominantly associated with reduced CT, with the strongest effects observed in the insula, auditory temporal regions, anterior and mid-cingulate cortex, and sensorimotor-related sulci (*P* < 0.01, *d* = −0.06 to −0.05; Fig. 5A and Table S1). Metabolomic signatures were predominantly linked to reduced FA in diverse projection, association, and limbic white-matter tracts (*P* < 0.001, *d* = −0.07 to −0.05; Fig. 5B and Table S2), spanning multiple segments of the internal capsule as well as the external capsule, superior fronto-occipital fasciculus, sagittal stratum, cingulum-hippocampus, fornix/stria terminalis, and bilateral cerebellar peduncles. Proteomic signatures were linked to reduced CV exclusively in the left short insular gyrus (*P* < 0.001, *d* = −0.12; Fig. 5C and Table S3). Although the 3 omic modalities showed uniformly negative associations with brain structural measures, each exhibited its spatial pattern in distinct structural domains. Additional modality-associated results are provided in Fig. S9 and Table S4.

### Association between the identified multi-omic signatures and polygenic risk scores

We evaluated the associations between the identified multi-omic signatures and polygenic risk scores (PRSs) for multiple diseases and traits to characterize their genetic architecture (Fig. 5D and Tables S11 to S13). The most extensive associations were observed for autoimmune disorders, including Crohn’s disease (CD; metabolomic: *d* = 0.03, *P =* 6.99 × 10^−11^), coeliac disease (CED; blood biochemistry: *d* = 0.01, *P =* 0.007; metabolomic: *d* = −0.02, *P =* 6.66 × 10^−4^; proteomic: *d* = −0.04, *P =* 8.11 × 10^−8^), psoriasis (PSO; metabolomic: *d* = 0.03, *P =* 3.74 × 10^−7^), type 1 diabetes (T1D; blood biochemistry: *d* = 0.03, *P =* 1.06 × 10^−6^; proteomic: *d* = −0.03, *P =* 1.69 × 10^−4^), systemic lupus erythematosus (SLE; metabolomic: *d* = −0.02, *P =* 1.07 × 10^−5^), multiple sclerosis (MS; blood biochemistry: *d* = −0.02, *P =* 0.002; metabolomic: *d* = −0.01, *P* < 0.05), and ulcerative colitis (UC; metabolomic: *d* = 0.01, *P* < 0.05). Depression PRS was significantly linked to both metabolomic and proteomic signatures (metabolomic: *d* = 0.04, *P* < 0.05; proteomic: *d* = 0.07, *P* < 0.05). Notably, the PRS for HDL cholesterol exhibited the strongest associations in blood cell counts and biochemistry (HDL; *d* = −0.29, *P* < 1 × 10^−6^) and metabolomics (*d* = −0.27, *P* < 1 × 10^−6^), and a weaker but significant effect in proteomics (*d* = −0.07, *P =* 3.47 × 10^−5^).

### Association between the identified multi-omic signatures and health-related phenotypes

To assess whether these signatures capture broader health variation, we examined their associations with 674 phenotypes across 9 domains. MCP-related signatures showed widespread associations with these phenotypes after Bonferroni correction: 278 with blood cell counts and biochemistry measures (|*d*| = 0.02 to 0.22; Fig. 5E and Table S5), 299 with metabolomics (|*d*| = 0.02 to 0.27; Fig. 5F and Table S6), and 150 with proteomics (|*d*| = 0.03 to 0.37; Fig. 5G and Table S7). The phenotypes showing the strongest associations with the MCP-derived multi-omic signatures were predominantly lifestyle, dietary, physical-activity traits, and several indicators of medical history.

Among lifestyle-related traits, smoking behavior showed the strongest associations with MCP-related multi-omic signatures. Pack-years of adult smoking as a proportion of lifespan exposed to smoking were positively associated with the identified signatures (blood biochemistry: *d* = 0.15, *n* = 34,514; metabolomic: *d* = 0.22, *n* = 34,514; proteomic: *d* = 0.20, *n* = 11,076). Dietary preference traits showed an opposite pattern. Stronger preferences for seafood, such as mackerel and salmon, were negatively associated with signatures (blood biochemistry: *d* = −0.17, *n* = 42,684; metabolomic: *d* = −0.17, *n* = 42,684; proteomic: *d* = −0.07, *n* = 13,080). Physical activity measures, such as the total number of active days, also showed negative associations (blood biochemistry: *d* = −0.13, *n* = 92,564; metabolomic: *d* = −0.14, *n* = 92,564; proteomic: *d* = −0.23, *n* = 28,378). Health and medical history indicators, such as a higher number of treatments or medications, were positively associated with the multi-omic signatures (blood biochemistry: *d* = 0.22, *n* = 115,595; metabolomic: *d* = 0.27, *n* = 115,595; proteomic: *d* = 0.37, *n* = 36,001).

## Discussion

**Fig. 6.**
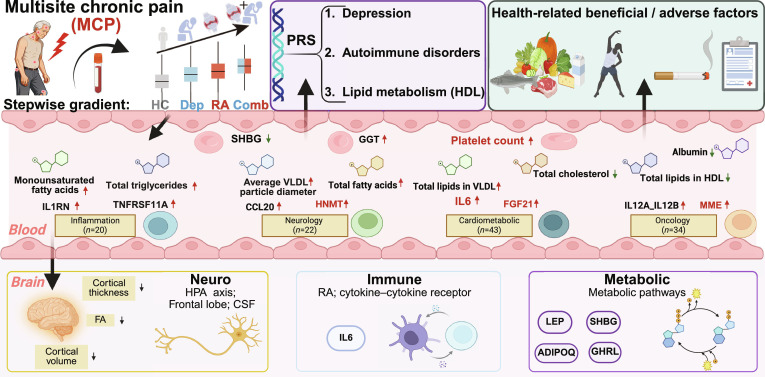
Integrative multidimensional framework linking depression and RA through MCP. Multi-omic association analyses revealed a stepwise gradient across disease states, with effect sizes increasing from depression to RA and being greatest in their comorbidity. Pathway enrichment, PPI, and PRS association analyses of the identified multi-omic signatures converged on neuro–immune–metabolic dysregulation. Analyses of health-related phenotypes revealed lifestyle and health-related factors associated with MCP-related multi-omic signatures, including fish intake and physical activity (negative associations) and smoking and indicators of medical burden (positive associations), suggesting potential relevance to pain burden in RA and depression. Together, this integrative multidimensional framework, encompassing both internal and external factors, provides a conceptual foundation for future mechanistic and therapeutic investigations.

This study integrated peripheral multi-omic data to delineate MCP-related molecular patterns underlying RA–depression comorbidity. These signatures exhibited graded dysregulation across increasing disease burden and partially mediated the bidirectional association between RA and depression. MR analyses supported causal effects of HNMT on depression risk and FGF21 on RA risk, identified MME as protective for depression, and highlighted platelet count, FGF21, and HNMT as shared causal factors. The signatures were further associated with modality-specific brain structural alterations, polygenic liability across immune, metabolic, and psychiatric traits, and health-related behaviors. Collectively, these findings position MCP as a systems-level phenotype indexing coordinated neuro–immune–metabolic dysregulation linking immune processes and depression (Fig. 6).

Clinically, RA and depression are heterogeneous disorders that frequently co-occur. Using data from more than 327,000 participants in the UK Biobank, we confirmed their bidirectional association: RA was associated with a 42% increased risk of depression, and baseline depression increased the risk of RA by 38%. MCP, a pain phenotype frequently observed in both RA and depression [11–14], provides a quantifiable indicator of broader systemic dysregulation. By linking MCP severity to blood-based molecular signatures, our findings outline a scalable approach for identifying shared peripheral features of RA and depression that may support early risk stratification for subsequent RA or depression onset. This is particularly relevant because rheumatology and psychiatry remain largely isolated in routine clinical practice, and shared molecular markers may help bridge this gap. These results also suggest a peripheral biological pathway that complements established brain–body models of RA–depression comorbidity [5,32,33]. From a clinical perspective, these findings suggest potential value for risk stratification in RA and depression. MCP-related multi-omic signatures may help identify individuals with greater disease burden who could benefit from closer monitoring and more coordinated care across rheumatology and psychiatry.

Most studies of RA–depression comorbidity have focused on single biological processes, particularly immune dysregulation [8], while the integrated multi-omic landscape remains largely underexplored. Using a supervised multi-omic integration framework with MCP as the reference phenotype [31], we identified shared multi-omic signatures that captured cross-omic covariation and strengthened associations between molecular components and MCP. These signatures were supported by prior research on pain, including evidence linking GGT and LEP to increased chronic pain risk and inverse associations observed for albumin and HDL [11,16]. GAST and RARRES2 have likewise been consistently observed across multiple pain sites [16]. Beyond their relevance to pain, these signatures also appear to be shared across RA and depression. Evidence from prior single-omic or single-disease studies has largely focused on isolated molecular features. For example, in depression, blood biochemical markers such as GGT has been linked to increased risk through immune activation [34], and proteomic markers such as HNMT has been implicated in histamine inactivation pathways [35]. In RA, dysregulated SHBG has been associated with disease susceptibility [36], while CCL20 has been identified as a key chemotactic mediator in inflammation [37]. Extending these single-omic findings, our multi-omic analysis showed that the MCP-related signatures were shared across RA and depression, encompassing molecular features across blood cell counts/biochemistry, metabolomics, and proteomics. Altered platelet counts in both conditions suggest shared inflammatory activity and involvement of neuroimmune interactions [38,39]. Elevated triglycerides and related metabolites point to disrupted lipid and energy metabolism, which is consistent with chronic low-grade inflammation and could, in turn, influence peripheral–central inflammatory signaling [7]. Proteomic alterations involving interleukin-6 (IL-6), TNFRSF11A, and IL1RN further implicate cytokine pathways central to joint pathology and broader neuroimmune processes relevant to pain and mood regulation [8]. These multi-layered disruptions support the view that RA and depression share interconnected physiological processes, where inflammation perturbs metabolic pathways, metabolic shifts feed back onto immune signaling, and cytokine activity shapes peripheral–central communication [7,8], rather than reflecting isolated pathway abnormalities.

We observed a significant linear trend in the identified molecular profiles across disease states, with effect sizes increasing from depression to RA and peaking in comorbidity, suggesting possible synergistic effects when both conditions co-occur. A plausible explanation is that both conditions involve elevated pro-inflammatory mediators [e.g., IL-6 and tumor necrosis factor-α (TNF-α)], neuroimmune activation, and metabolic disturbances [7]. The co-occurrence of these conditions may exert additive or synergistic effects that intensify molecular damage. This interpretation aligns with clinical evidence linking comorbid RA and depression to increased pain and mortality risks [6,7]*.* Collectively, these findings indicate that individuals with comorbid RA and depression may experience a heightened biological burden, emphasizing the need for targeted clinical assessment and management in this population.

The identified multi-omic signatures were associated with an increased risk of developing RA and depression and explained 1.67% to 9.64% of the bidirectional association. Although modest, these mediating effects remained statistically significant after adjustment for multiple confounders, suggesting a partial mediating role in the relationship between RA and depression. However, MR analyses indicated causal relationships for only a subset of these signatures with either condition. These causal factors encompassed both well-established immune mediators (e.g., IL6 and platelet count) and less-characterized molecules (e.g., HNMT and KLK1) [38–41]. Among these causal associations, FGF21 emerged as a causal risk factor for RA. This finding aligns with elevated circulating and synovial FGF21 levels and their correlation with inflammatory activity [42]. HNMT was identified as a putative causal risk factor for depression, consistent with its role in regulating central histamine clearance and its involvement in neuropsychiatric processes [35]. Conversely, MME exhibited a protective effect on depression. Prior studies reporting its co-expression across depression and metabolic disorders suggest potential involvement in shared biological pathways [43]. Notably, 3 peripheral signatures, including HNMT, FGF21, and platelet count, were detected in both RA- and depression-related MR analyses. These molecules represent distinct yet interconnected biological domains, including inflammatory signaling (platelet count) [38,39], metabolic signaling involving FGF21 [42], and neuropsychiatric processes related to HNMT [35]. The convergence of these signals across conditions highlights potential shared neuro–immune–metabolic dysregulation in RA–depression comorbidity.

Prior studies have repeatedly reported structural alterations, specifically reduced insular volume [44] and cortical thinning in the insula and anterior cingulate cortex [45], regions that play key roles in pain perception and emotion regulation and are commonly implicated in RA-related chronic pain and depressive symptoms [45–47]. In addition, our observations align with prior diffusion MRI work showing widespread FA reductions across chronic pain cohorts, including notably lower FA in the anterior limb of the internal capsule among individuals with RA and comorbid depression [48–50]*.* Our analyses further showed that different omic layers exhibited distinct patterns of association with neuroimaging modalities: Biochemical markers displayed their broadest associations with CT, metabolomic signatures with white-matter microstructure, and proteomic signatures with CVs. These modality-specific associations indicate that each omic layer captures physiological processes reflected in different neuroanatomical domains, reinforcing the value of integrating multiple omic layers to obtain a more comprehensive characterization of MCP-related neurobiology [51]. It is worth noting that the observed effect sizes were modest, although statistically significant after correction for multiple comparisons, consistent with prior large-scale neuroimaging studies indicating that such associations are typically small [52,53].

The identified multi-omic signatures showed extensive associations with PRSs across multiple autoimmune disorders, suggesting that the captured polygenic liability reflects a broader autoimmune diathesis. This view aligns with evidence that a substantial proportion of RA risk loci exhibit pleiotropy with other immune-related diseases, reflecting shared genetic architecture across these conditions [54]. The comparatively weaker associations observed for RA PRS may reflect that, although RA represents a central clinical focus of this study, the identified multi-omic signatures capture broader systemic and immune-related processes that extend beyond RA-specific genetic risk. Strong PRS associations with lipid metabolism and depression indicated that shared genetic influences across immune and metabolic traits may contribute to the links among RA, depression, and chronic pain. Beyond genetic correlates, the MCP-related signatures were associated with a range of health-related phenotypes spanning 9 domains. Current smoking was linked to greater pain burden, whereas oily fish consumption and physical activity were associated with reduced burden, consistent with epidemiological evidence [55,56]. These behaviors co-varied with the multi-omic signatures and promoted systemic inflammation and metabolic dysregulation, potentially increasing vulnerability to both RA and depression [57–61]. Together, these observations highlight behavioral domains that may help explain the overlapping pain burden of RA and depression.

Our study has several limitations. First, pain phenotypes were self-reported using a questionnaire that, although widely used, did not capture information on pain duration or intensity. Most studies, including ours, have focused on the number of pain sites, whereas specific co-occurring pain patterns have received less attention [62]. For example, RA may present with localized symmetrical joint pain or with widespread pain [6], highlighting the need to assess how pain patterns shape RA–depression comorbidity. Second, although we examined disease heterogeneity using complementary dimensions (e.g., MCP burden and disease status), depression and RA were each treated as relatively homogeneous groups based on available ICD-coded classifications. This approach may overlook important within-disease heterogeneity. Future studies incorporating more detailed clinical phenotyping will be needed to investigate subtype-specific multi-omic mechanisms. Third, the identified multi-omic signatures were derived primarily from peripheral blood samples, which may not capture tissue-specific processes such as synovial alterations in RA [7]. To further inform interpretation, we annotated the tissue specificity of the identified proteins using data from the Human Protein Atlas [63] (Table S14); however, this approach does not fully resolve tissue-specific effects. Future integration of large-scale proteomic data from tissue-specific sources and cerebrospinal fluid will provide deeper mechanistic insights. Fourth, although multiple sensitivity analyses supported a graded pattern across disease burden, these findings should be interpreted with caution given the relatively small sample size of the comorbidity group and require replication in larger cohorts. In addition, the psychiatric dimension of this study is limited to depression, and whether these findings extend to other psychiatric disorders warrants further investigation. Finally, the use of the predominantly European UK Biobank cohort may limit the generalizability of our findings to more diverse populations. The limited availability of independent cohorts with comparable multi-omic measurements and detailed pain, RA, and depression phenotyping restricted our ability to comprehensively assess replication across studies. Future large-scale studies with harmonized multi-omic platforms and standardized phenotypic definitions will be essential to validate and extend our findings.

In summary, we identify peripheral multi-omic signatures reflecting coordinated neuro–immune–metabolic dysregulation underlying MCP-associated comorbidity between RA and depression. These signatures link to brain structural variation, genetic susceptibility, and behavioral factors, positioning MCP as a potential biological nexus connecting immune processes and depression. This integrative framework may help clarify mechanisms of immune–depression comorbidity and guide more targeted approaches to reducing pain-related disease burden.

## Materials and Methods

### Study design and population

The UK Biobank is a large-scale prospective cohort study involving over 500,000 individuals aged 37 to 73 years [64]*.* Ethical approval was granted by the North West Multi-center Research Ethics Committee (11/NW/0382), and all participants gave written informed consent. Recruitment was conducted between 2006 and 2010 at 22 assessment centers, where participants completed a touchscreen questionnaire and a face-to-face interview, underwent physical measurements, and provided biological samples.

For this study, participants were categorized into 6 groups: (a) healthy controls (participants without any mental disorders or immune-related arthritis at baseline assessment, *N* = 5,605). For sensitivity analyses, we further excluded participants with other major medical conditions, following the criteria described by Tian et al. [65], resulting in a conservative subset (*N* = 3,605); (b) a prevalent depression group (individuals with depression at or before the baseline assessment, *N* = 7,565); (c) a prevalent RA group (individuals with RA at or before the baseline assessment, *N* = 1,029); (d) an RA–depression comorbidity group (diagnosed with both depression and RA at or before the baseline assessment; *N* = 120; (e) a future-onset depression group (incent cases during follow-up, *N* = 3,963); and (f) a future-onset RA group (incent cases during follow-up, *N* = 1,202). Detailed criteria for participant selection were presented in Fig. S1. Detailed demographic characteristics of all study cohorts are provided in Table S15.

### Depression and RA diagnoses

Information on the prevalence and incidence of depression and RA was obtained from the UK Biobank “First Occurrence” category (category 1712), which was derived from the integration of hospital admissions, death records, primary care data, and self-reported outcomes collected during nurse-led interviews. Depression was defined as ICD-10 codes F32 (depressive episode) or F33 (recurrent depressive disorder), while RA was defined as ICD-10 codes M05 (seropositive RA) or M06 (other RA). These definitions were based on ICD-coded classifications and were not further subdivided into clinical subtypes. Comorbidity of depression and RA was defined as the presence of both conditions at baseline. Participants were followed from baseline until the earliest occurrence of the outcome of interest, death, loss to follow-up, or censoring.

### MCP assessment

Pain conditions were evaluated via a touchscreen questionnaire asking whether participants had experienced any activity-interfering pain in the past month. Options included pain at back, facial, head, knee, stomach/abdomen, hip, neck/shoulder, all over the body, or none of the above. For each selected pain type, participants reported whether it had lasted more than 3 months. Pain lasting longer than 3 months was classified as chronic pain, consistent with the International Association for the Study of Pain definition. A chronic pain site score (range, 0 to 7) was calculated accordingly. As per previous studies [66], participants with pain all over the body were excluded from calculating the pain site number due to the lack of site-specific information. In all primary analyses, the MCP score was modeled as a continuous variable to capture the graded nature of pain burden.

### MCP-related multi-omic signatures

UK Biobank developed a comprehensive multi-omic dataset by analyzing baseline blood samples from participants. This dataset comprised blood cell counts and biochemistry, NMR-based metabolites, and proteomic profiles using the Olink platform. Quality control procedures and further methodological details were described in the corresponding UK Biobank documentation: https://biobank.ndph.ox.ac.uk/showcase/showcase/docs/biomarker_issues.pdf, https://biobank.ndph.ox.ac.uk/ukb/ukb/docs/nmrm_companion_doc.pdf, and https://biobank.ndph.ox.ac.uk/ukb/ukb/docs/PPP_Phase_1_QC_dataset_companion_doc.pdf.

The multi-omic data were categorized into 3 domains: (a) blood cell counts and biochemistry: a total of 61 measurements were obtained, encompassing hematological parameters and biochemical signatures. Due to limited coverage and the consequent loss of statistical power, estradiol and the rheumatoid factor were excluded from our primary analyses. These 2 signatures were analyzed separately (Fig. S6). (b) NMR metabolomics: this included 168 directly measured metabolic biomarkers. (c) Proteomics: a total of 2,923 unique proteins were measured across 4 Olink panels (cardiometabolic, inflammation, neurology, and oncology). Normalized protein expression (NPX) values provided by Olink were utilized (Supplementary Methods 1). Following previous studies, proteins with >60% missing data (*n* = 3) were excluded, and median imputation was applied to the remaining missing values, yielding 2,920 proteins for analysis. Additional details of quality control procedures across all omic layers are provided in Supplementary Methods 1.

### Multi-omic fusion with reference: MCP

To identify multi-omic molecular components associated with the MCP score, we performed an MCP-guided supervised 3-way integration in healthy controls (*N* = 5,605). Specifically, MCP scores were used as a reference to jointly decompose peripheral datasets, including blood cell counts and biochemistry ($X_{1}$; *N* × 59 matrix), metabolomic profiles ($X_{2}$; *N* × 168 matrix), and proteomic profiles ($X_{3}$; *N* × 2,920 matrix), using MCCAR (multimodal canonical correlation analysis with reference) [31,67]*.* Each omic layer $X_{k}$ was modeled as a linear mixture of components $S_{k}$ with a nonsingular mixing matrix $A_{k}$ (*k* = 1, 2, 3), namely, $X_{k}=A_{k}\times S_{k}$. The MCCAR algorithm imposes an additional constraint to maximize not only cross-omic covariation but also the correlations between each omic layer and the MCP reference variable, a strategy that has been widely applied in previous neuroimaging studies across both psychiatric and nonpsychiatric conditions [29,30]*.* This approach was chosen because it allows incorporation of an external reference variable (MCP) to guide component extraction in a supervised manner while capturing shared variation across multiple omic layers. The optimization objective is formulated in Eq. (1):

$$\max\sum_{k,j=1}^{3}\left\{{\left\|\text{corr}\left(A_{k},\;A_{j}\right)\right\|}_{2}^{2}+2\lambda {\left\|\text{corr}\left(A_{k},\;\mathrm{MCP}\right)\right\|}_{2}^{2}\right\}$$ (1)

Here, *corr* ($A_{k}$, $A_{j}$) represents column-wise correlations between omic layers, and co*rr*($A_{k}$, MCP) denotes their correlations with the MCP vector (*N* × 1). The regularization parameter λ balances cross-omic covariance and reference alignment. The optimal value (λ = 0.5) was determined using repeated 5-fold cross-validation to maximize the stability and reproducibility of the identified components (see Fig. 10). After optimization, MCCAR yielded omic-specific components $\;S_{k}=A_{k}^{-1}X_{k}$ that are maximally correlated across omic layers and with the MCP scores. The analyses were implemented using the Fusion ICA Toolbox (FIT; https://trendscenter.org/software/fit).

The resulting multimodal signatures were linearly projected onto patient groups to obtain subject-wise weights $\;A_{k}^{\text{patients}}$ for individuals with prevalent depression (*N* = 7,565), prevalent RA (*N* = 1,029), RA–depression comorbidity (*N* = 120), future-onset depression (*N* = 3,963), and future-onset RA (*N* = 1,202), as in Eq. (2):

Akpatients=XkpatientsSk−1 (2)

This projection enabled group-level comparisons within MCP-linked multi-omic signatures to investigate the molecular mechanisms underlying the shared pathophysiology of RA and depression. Pearson correlations were calculated between MCP scores and cross-omic component weights $\left(A_{k}^{\text{patients}}\right)$. Group differences in the identified multi-omic component weights were evaluated using one-way ANOVA with polynomial trend analysis, complemented by 2-sample *t* tests for comparisons between each patient group $\left(A_{k}^{\text{patients}}\right)$ and healthy controls ($A_{k}$).

Each omic-layer component $S_{k}$ was standardized into *Z* scores and thresholded at |*Z*| > 2 to extract MCP-related molecular signatures. Note that, prior to data fusion, potential confounding effects of age, sex, batch, season, and fasting duration at blood collection, duration between blood collection and protein measurement (as determined by the plate and panel for each participant and protein to account for plate effects), and the top 20 genetic principal components were regressed out from each omic dataset (see Supplementary Methods 2).

### Associations with the incidence of depression and RA

We used Cox proportional hazards models to study the bidirectional association between depression and RA using follow-up time as the underlying timescale. Building on this framework, we further assessed whether MCP-related signatures from each omic layer were prospectively associated with incident depression or RA, with age stratified by quartiles to address violations of the proportional hazards assumption. We adjusted for age, sex, BMI, alcohol consumption, smoking status, ethnicity, household income, education level, deprivation index, and metabolic syndrome in all models (details provided in Supplementary Methods 2). Multiple testing correction was applied using the FDR method.

The proportional hazards assumption was evaluated using both scaled Schoenfeld residuals and rank-based tests [68], with no consistent or global violations observed. Multicollinearity among covariates was low across all models (variance inflation factors, VIFs < 1.8). To address reverse causation, participants developing RA or depression within 2 years of follow-up after baseline were excluded in a sensitivity analysis*.*

### Mediation analyses

Mediation analyses were conducted using the mediation package in R to evaluate whether the identified multi-omic signatures mediate the bidirectional associations between depression and RA. Two separate mediation models were constructed: (a) The first model tested whether the identified multi-omic signatures explained the association between baseline RA status and subsequent risk of depression; (b) the second model examined whether the identified multi-omic signatures explained the association between baseline depression status and subsequent risk of RA. The significance of mediating effects was assessed using 5,000 bootstrap iterations. All analyses were conducted in R 4.4.1, adjusted for the same set of covariates, and corrected for multiple comparisons using FDR correction.

### Causal inference using MR

We conducted bidirectional 2-sample MR analyses to evaluate the potential causal effects of genetically predicted MCP-related multi-omic signatures on depression and RA, using the TwoSampleMR package in R [69]*.* Summary statistics of genome-wide association study (GWAS) for depression, RA, and the identified multi-omic signatures (119 proteins, 8 metabolites, and 3 blood cell counts and biochemistry traits) were obtained from independent datasets (detailed sources in Table S10). Following prior studies [70], genome-wide significant SNPs (*P* < 5 × 10^−8^) for each exposure were clumped based on linkage disequilibrium (*r*^2^ < 0.01; 1000 Genomes Project, European ancestry) and harmonized to align effect alleles. The IVW method was used as the primary approach for MR analysis, and the weighted median, simple median, and MR-Egger methods were used as complementary approaches. Heterogeneity was assessed using Cochran’s *Q* test, and a multiplicative random-effects IVW model was used when significant*.* Horizontal pleiotropy was assessed via the MR-Egger intercept, and outliers were identified and corrected using the MR-PRESSO*.* Finally, leave-one-SNP-out analyses were performed to evaluate the influence of single SNP on causal estimates [71]*.*

### Functional enrichment analysis and PPI network

Functional enrichment analyses and PPI network construction were performed using the STRING database (v11.5) [72], which integrates both experimentally validated and computationally predicted protein interactions. Proteins positively and negatively associated with MCP were analyzed separately. Enrichment was conducted across multiple annotation sources, including Gene Ontology (GO) categories: Biological Processes, Cellular Components, and Molecular Functions, Kyoto Encyclopedia of Genes and Genomes (KEGG) pathways, tissue-specific expression profiles, and disease–gene associations. Terms with FDR < 0.05 were considered statistically significant. PPI networks were also generated and visualized using Cytoscape (v3.10.2) [73].

### Association between MCP-related multi-omic signatures and brain structure

Structural and diffusion MRI data were processed by the UK Biobank imaging team and released as imaging-derived phenotypes (IDPs) to approved researchers. Detailed pipelines are described in the UK Biobank Brain Imaging Documentation and related publications [74]. The IDPs analyzed in this study included CT and CV across 148 cortical regions [75], and tract-averaged FA across 48 standard-space white-matter tracts [76].

We employed a linear mixed-effect model to examine how the identified multi-omic signatures relate to each of the included brain measures. The identified signatures from each omic layer were modeled separately as fixed effects, with the UK Biobank assessment center modeled as a random effect. Each brain measure (CT, CV, and FA) was modeled as the dependent variable. Models were adjusted for the previously described covariates and intracranial volume, and multiple comparisons across brain regions were corrected using the FDR method.

### Association between MCP-related multi-omic signatures and PRSs

We also explored the associations between multi-omic signatures and PRSs for a wide range of physical traits and diseases from the UK Biobank using linear regression models. Depression PRS was not available in UK Biobank and was manually computed in-house using imputed genotype data following standard quality control (QC; see Supplementary Methods 3 for details). The identified signatures from each omic layer were entered separately as independent variables, with each PRS analyzed as a dependent variable in separate models. All models were adjusted for the same covariates described previously. Multiple testing correction was applied using the FDR.

### Association between MCP-related multi-omic signatures and health-related phenotypes

We examined associations between multi-omic signatures and 674 health-related phenotypes, which were grouped into 9 broad domains: cognitive function, lifestyle factors, dietary habits and food preferences, working and living environment, physical measures, health and medical history, physical activity, mental health, and ophthalmologic traits (Table S5). Most phenotypes were self-reported via touchscreen questionnaires at assessment centers or online surveys. Regression models were selected according to the type of outcome variable. Linear regression was employed for continuous and ordinal categorical traits, while logistic regression was used for binary traits. All models were adjusted for covariates described previously. To facilitate effect size comparisons across different outcome types, regression coefficients (β) for continuous traits and log-transformed ORs for binary traits were converted to Cohen’s *d* using standard procedures (the effect size package in R v4.1.2) [77]. Given the relatively large number of comparisons (*n* = 674), Bonferroni correction was applied to obtain more conservative results, setting the significance threshold at *P* < 0.05/674.

## Ethical Approval

This study used data from the UK Biobank. The UK Biobank study was approved by the North West Multicenter Research Ethics Committee (no. 11/NW/0382), and all participants provided written informed consent. The present analyses were conducted under the standard UK Biobank access procedure. For publicly available summary-level data from FinnGen, ethical approval and informed consent had been obtained in the original studies.

## Data Availability

The data used in this study were obtained from the UK Biobank under an approved application and are available through the UK Biobank Access Management System (https://www.ukbiobank.ac.uk/enable-your-research/apply-for-access). Software packages used in this study include R v4.4.1 with the survival, survminer, and mediation packages (https://www.r-project.org), TwoSampleMR v2.0, STRING (STRING: functional protein association networks), Cytoscape v3.10.2 (https://cytoscape.org), Fusion ICA Toolbox (https://trendscenter.org/software/fit), MATLAB R2020b (https://www.mathworks.com/products/matlab.html), BrainNet Viewer v20191031 (https://www.nitrc.org/projects/bnv), Python v3.8.13 (https://www.python.org), and ggplot2 v3.4.2 (https://ggplot2.tidyverse.org). The analysis code used in this study is available at https://github.com/qianwang-brain/Pain_RheumatoidArthritic_Depression_Fusion.
