# Supplementary material for: Multisite Chronic Pain Reveals Neuro–Immune–Metabolic Dysregulation across Rheumatoid Arthritis and Depression
Source: Research (Wash D C). 2026 Jun 17;9:1298. doi: 10.34133/research.1298 (PMC13272895; doi:10.34133/research.1298)
Supplement: Supplementary 1 — Figs. S1 to S11 Tables S1 to S18 [file research.1298.f1.zip › Supplementary_information.docx]

Supplementary Materials

Supplementary methods:

Supplementary Methods 1: Quality control of multi-omic data

Supplementary Methods 2: Covariates

Supplementary Methods 3: PRS for major depressive disorder

**References**

Supplementary figures:

*Supplementary Figure 1*. Flowchart illustrating criteria for participant selection.

*Supplementary Figure 2*. MCP-related multi-omics fusion results in the stricter healthy control subset (N = 3,605).

*Supplementary Figure 3*. Correlations between the identified multi-omic signatures and MCP.

*Supplementary Figure 4.* Sensitivity t-test analyses between patient and control groups at baseline using 1,000 bootstrap iterations.

*Supplementary Figure 5.* Sensitivity t-test analyses between future patient and control groups, based on 1,000 bootstrap iterations.

*Supplementary Figure 6*. Rheumatoid factor and oestradiol analyses.

*Supplementary Figure 7.* Prospective and causal associations between identified multi-omic signatures and incident depression or RA after exclusion of cases occurring within two years of baseline.

*Supplementary Figure 8.* Leave-one-SNP-out analyses.

*Supplementary Figure 9.* Additional analyses: associations between the identified multi-omic signatures and brain structure.

*Supplementary Figure 10.* Parameter tuning of the regularization parameter λ in MCCAR based on repeated cross-validation.

*Supplementary Figure 11.* Robustness of linear gradient to group size imbalance across omic layers.

**Supplementary Methods 1: Quality control of multi-omic data**

Quality control procedures for blood cell counts and biochemistry as well as NMR-based metabolomic data were implemented by the UK Biobank and are described in the corresponding documentation (see UK Biobank documentation for details: https://biobank.ndph.ox.ac.uk/showcase/showcase/docs/biomarker_issues.pdf; https://biobank.ndph.ox.ac.uk/ukb/ukb/docs/nmrm_companion_doc.pdf; https://biobank.ndph.ox.ac.uk/ukb/ukb/docs/PPP_Phase_1_QC_dataset_companion_doc.pdf)In this study, additional quality control procedures were applied to the proteomic data, as described below.

Proteomic data were subjected to stringent quality control procedures, as detailed in the Supplementary Information of Sun et al.*^1^* Protein abundances were reported as Normalized Protein eXpression (NPX) values, following log transformation and normalization both within and across batches. Additional quality control was performed by the UK Biobank Pharma Proteomics Project, which excluded outliers and data flagged with quality control warnings by Olink. No further processing of NPX values was undertaken after these steps.

Among the 55,323 participants with available proteomic measurements, a total of 2,923 unique proteins were quantified. Of these, 53,026 participants with baseline measurements were initially included in the analysis. Participants with more than 60% missing protein data, as well as proteins absent in over 60% of individuals, were excluded. After applying these filters, the final dataset comprised 52,632 participants and 2,920 proteins.

**Supplementary Methods 2: Covariates**

Covariates were derived as follows. Age was calculated from date of birth and the date of baseline assessment (Field 21003). Sex (Field 31) and ethnicity (Field 21000) were self-reported. Batch (Field 30901), assessment centre (Field 54), season and fasting duration at blood collection (Field 74), and the time interval between blood sampling (Field 3166) and protein measurement were included to account for technical and temporal variation. The top 20 genetic principal components (Field 22009) were incorporated to control for population structure, and plate-specific effects were adjusted for according to the plate and panel for each participant and protein. Ethnicity was dichotomised as White versus non-White; participants selecting “prefer not to answer” or “do not know” (<0.5%) were classified as non-White. Body mass index was calculated as weight divided by height squared (kg m⁻²). Body mass index (BMI; Field 21001) was calculated as weight (kg) divided by height squared (m²). Socioeconomic status was derived from residential postcodes using Townsend deprivation scores (Field 189) and categorized into tertiles. Educational attainment (Field 6138) was dichotomized as having a university/college degree versus not. Household income (Field 738) was categorized as low (<£52,000), middle (£52,000–£100,000), or high (>£100,000). Participants who declined to report income (9.4%) were retained as a separate “unknown” category to preserve statistical power. Smoking status (Field 20116) was categorized as never versus ever. Alcohol intake frequency (Field 1558) was modeled as an ordinal variable. Metabolic syndrome was defined as the presence of ≥3 of the following components: (1) central obesity (waist circumference >88 cm in women or >102 cm in men); (2) elevated glycemia/diabetes (fasting glucose ≥5.6 mmol/L or self-reported physician diagnosis); (3) hypertension (systolic blood pressure ≥130 mmHg, diastolic blood pressure ≥85 mmHg, or self-reported physician diagnosis); (4) hypertriglyceridemia (≥1.7 mmol/L); and (5) low HDL cholesterol (<1.3 mmol/L in women or <1.0 mmol/L in men).

***Supplementary Methods 3*: PRS for major depressive disorder**

Genotype data for all 500,000 UK Biobank participants were available, with genotyping and initial quality control performed by the UK Biobank.^2^ Because PRS for major depressive disorder were not provided by the UK Biobank, we derived them manually using PRS-CS,^3^ a Bayesian framework that integrates GWAS summary statistics with an external linkage disequilibrium (LD) reference panel to estimate SNP effect sizes under a continuous shrinkage prior. GWAS summary statistics for major depressive disorder were obtained from the Psychiatric Genomics Consortium.^4^

Before PRS construction, stringent quality control was applied to the UK Biobank SNP data, excluding variants with missingness >0.05, minor allele frequency (MAF) <0.01, or deviation from Hardy–Weinberg equilibrium (p < 1×10⁻⁶). At the sample level, individuals with genotype missingness >0.05 or heterozygosity ±3 standard deviations from the mean were also removed. The resulting PRSs were standardized to a mean of zero and standard deviation of one, and subsequently used as predictors in downstream analyses.

**References**

1. Sun, B. B. *et al.* Plasma proteomic associations with genetics and health in the UK Biobank. *Nature* **622**, 329–338 (2023).

2. Bycroft, C. *et al.* The UK Biobank resource with deep phenotyping and genomic data. *Nature* **562**, 203–209 (2018).

3. Ge, T., Chen, C.-Y., Ni, Y., Feng, Y.-C. A. & Smoller, J. W. Polygenic prediction via Bayesian regression and continuous shrinkage priors. *Nat Commun* **10**, 1776 (2019).

4. Jiang, R. *et al.* The brain structure, inflammatory, and genetic mechanisms mediate the association between physical frailty and depression. *Nat Commun* **15**, 4411 (2024).


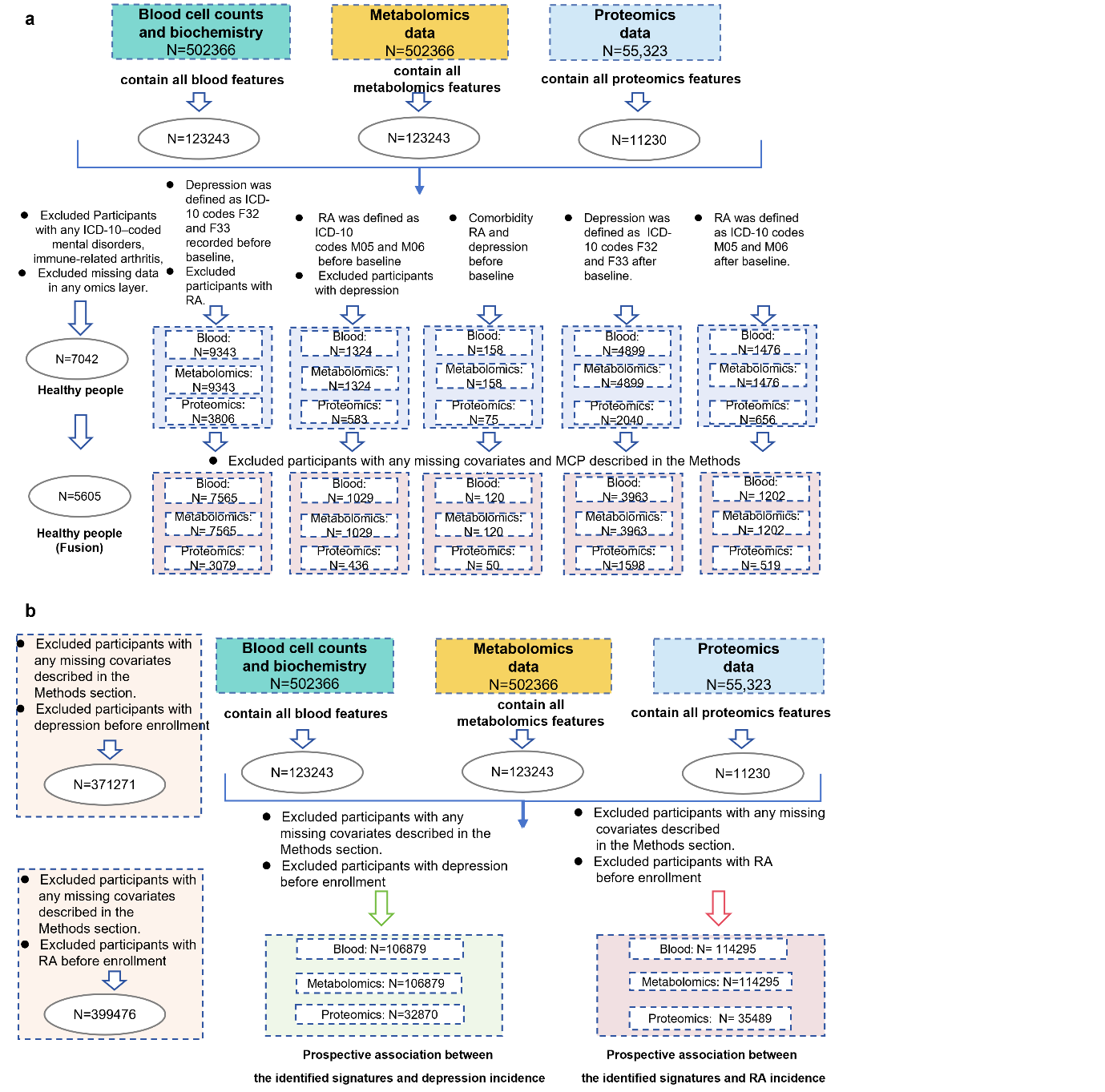


***Supplementary Figure 1*.** Flowchart illustrating the criteria for participant selection. **a,** Flowchart showing the criteria for sample selection for MCCAR analyses. **b,** Flowchart showing the criteria for sample selection for prospective and mediation analyses conducted in the present study.

**_
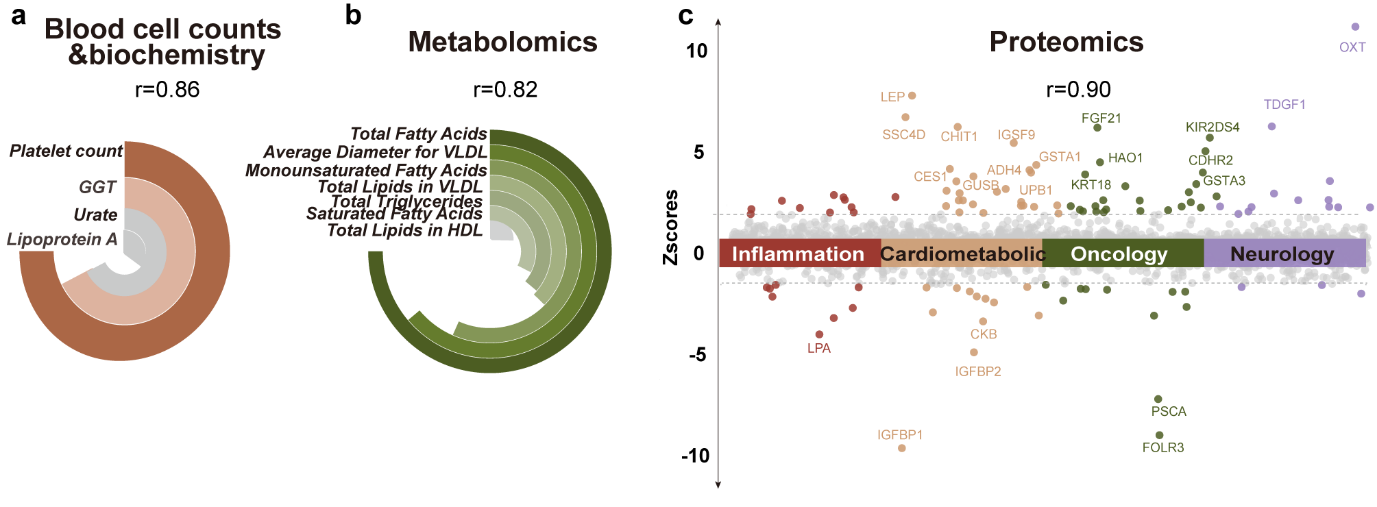
_*Supplementary Figure 2*. MCP-related multi-omics fusion results in the stricter healthy control subset (N = 3,605).** a, Four MCP-related signatures were identified in the blood cell counts and biochemistry modality (|Z| > 2), showing a correlation of r = 0.86 with the main results. B, Seven MCP-related signatures were identified in the metabolomics modality (|Z| > 2), with a correlation of r = 0.82 with the main results. C, A total of 108 signatures were identified in the proteomics modality, showing a correlation of r = 0.90 with the main results. The Manhattan plot showed the spatial distribution of 2,920 plasma proteins, with non-significant proteins (|Z| < 2) displayed in gray. The reported correlations (r) were computed using Pearson correlation across the union of significant signatures identified in both fusion analyses.

**
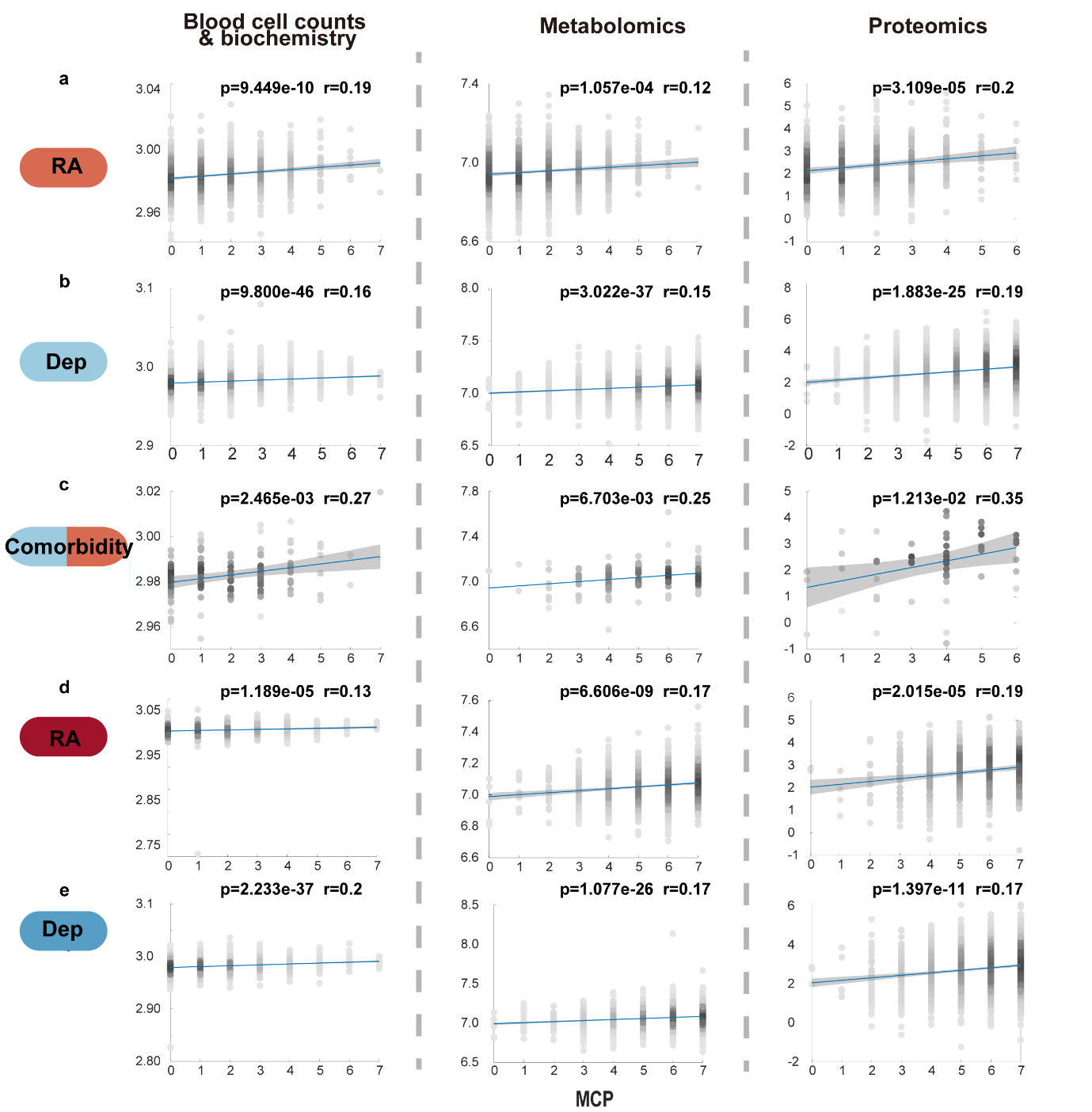
**

***Supplementary Figure 3*. Correlations between the identified multi-omic signatures and MCP. a,** In patients with RA, MCP was positively correlated with blood biochemistry (r = 0.19, p = 9.45 × 10⁻¹⁰), metabolomics (r = 0.12, p = 1.06 × 10⁻⁴) and proteomics (r = 0.20, p = 3.11 × 10⁻⁵). **b,** For depression, MCP showed positive correlations with blood biochemistry (r = 0.16, p = 9.80 × 10⁻⁴⁶), metabolomics (r = 0.15, p = 3.02 × 10⁻³⁷) and proteomics (r = 0.19, p = 1.88 × 10⁻²⁵). **c,** In the comorbid group, MCP was positively associated with blood biochemistry (r = 0.27, p = 2.47 × 10⁻³), metabolomics (r = 0.25, p = 6.70 × 10⁻³) and proteomics (r = 0.35, p = 1.21 × 10⁻²). **d,** In individuals at risk of future RA, MCP was positively correlated with blood biochemistry (r = 0.13, p = 1.19 × 10⁻⁵), metabolomics (r =0.17, p = 6.61 × 10⁻⁹) and proteomics (r = 0.19, p = 2.02 × 10⁻⁵). **e,** Finally, in individuals at risk of future depression, MCP exhibited a positive correlation with blood biochemistry (r = 0.20, p = 2.23 × 10⁻³⁷), metabolomics (r = 0.17, p = 1.08 × 10⁻²⁶) and proteomics (r = 0.17, p = 1.40 × 10⁻¹¹).

**
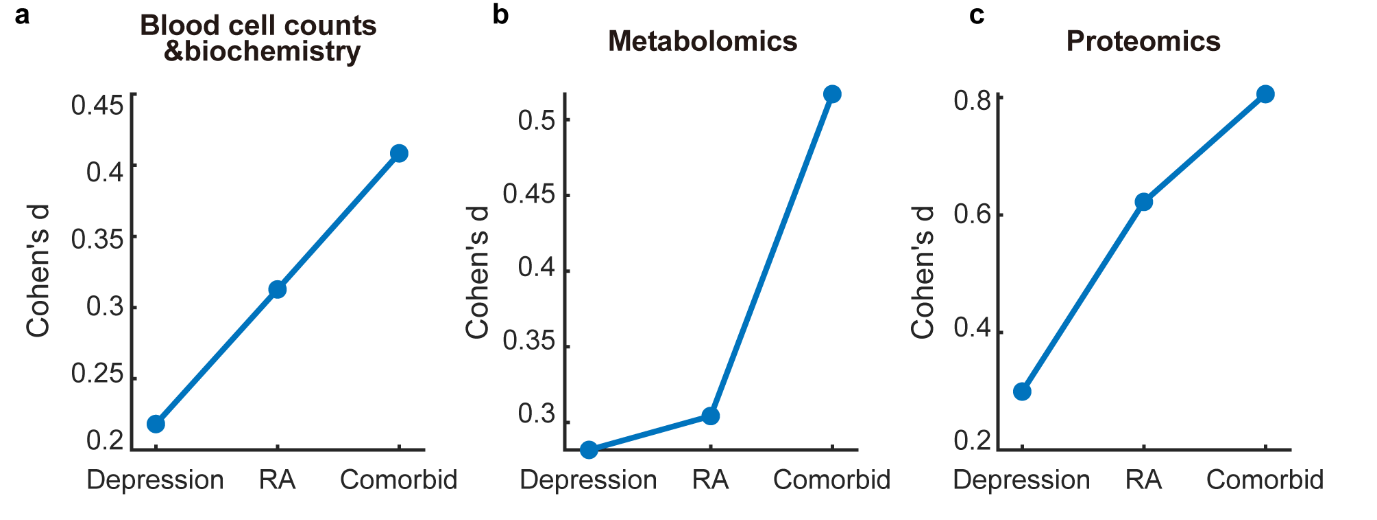
**

***Supplementary Figure 4*.** **Sensitivity *t*-test analyses between patient and control groups at baseline using 1,000 bootstrap iterations. a,** In blood cell counts / biochemistry, the mean magnitude of case–control differences progressively increased from the depression group (Cohen’s d = 0.24, p = 2.55× 10⁻^39^) to the RA group (d = 0.34, p = 9.71 × 10⁻^11^), with the largest effect observed in the comorbid group (d =0.43, p = 7.32 × 10⁻^3^). **b,** A similar pattern was observed in metabolomics, where mean effect sizes increased from depression (d = 0.28, p = 2.65 × 10⁻^52^) to RA (d = 0.49, p = 1.04 × 10⁻^21^), reaching the greatest magnitude in the comorbid group (d = 0.52, p = 7.77 × 10⁻^4^). **c,** In proteomics, mean group differences followed the same trend, progressing from depression (d = 0.30, p = 4.02 × 10⁻^25^) to RA (d =0.48, p = 3.06 × 10⁻^8^), and were most pronounced in the comorbid group (d = 0.81 p = 9.37 × 10⁻^4^).

**
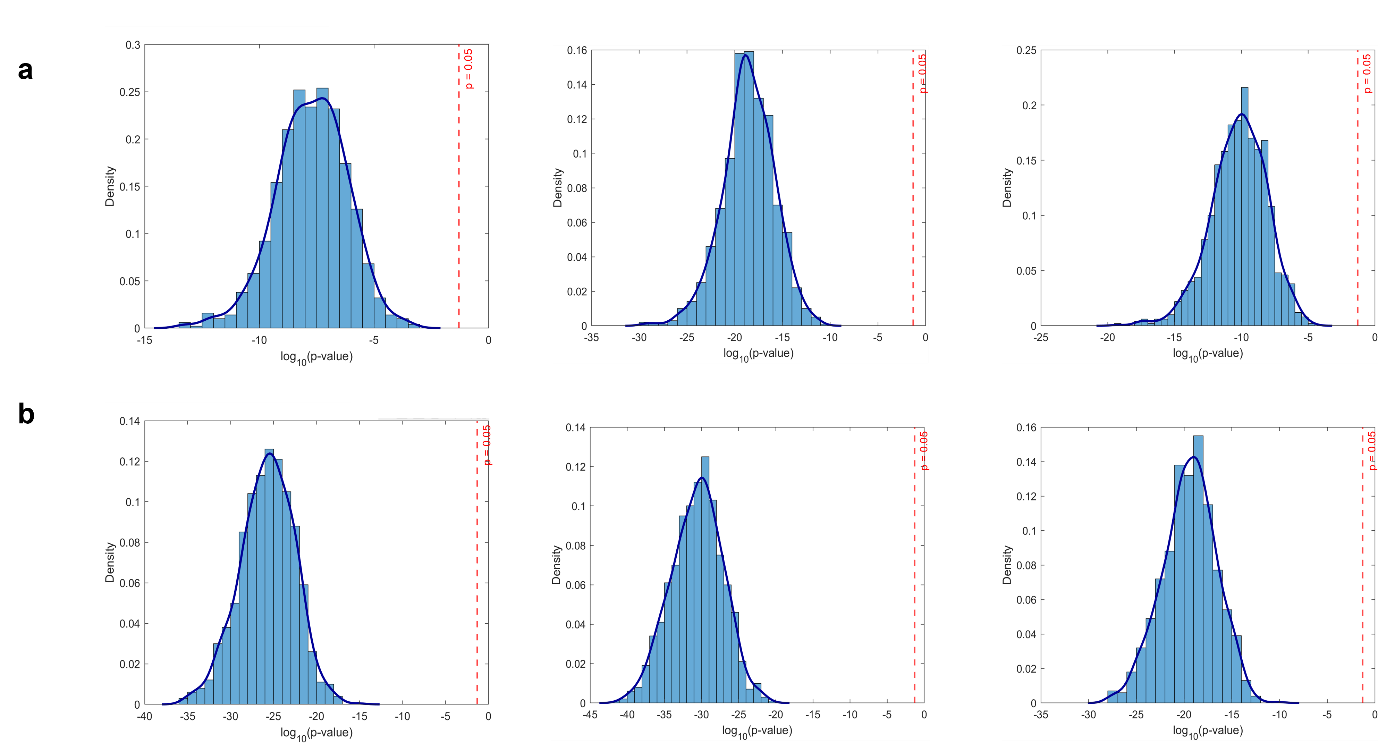
**

***Supplementary Figure 5*. Sensitivity t-test analyses between future patient and control groups, based on 1,000 bootstrap iterations.** **a,** Significant group differences were observed between healthy controls and individuals who later developed RA, with mean effect sizes indicating alterations in blood biochemistry (d = 0.23, p = 2.85 × 10⁻^6^), metabolomics (d = –0.37, p = 2.93 × 10⁻^14^), and proteomics (d = –0.41, p = 6.36 × 10⁻^8^). **b,** Similar differences were identified in individuals who subsequently developed depression, with consistent abnormalities across blood biochemistry (d = 0.24, p = 6.41 × 10⁻^19^), metabolomics (d = –0.26, p = 6.36 × 10⁻^8^), and proteomics (d = –0.33, p = 7.58 × 10⁻^14^).

**
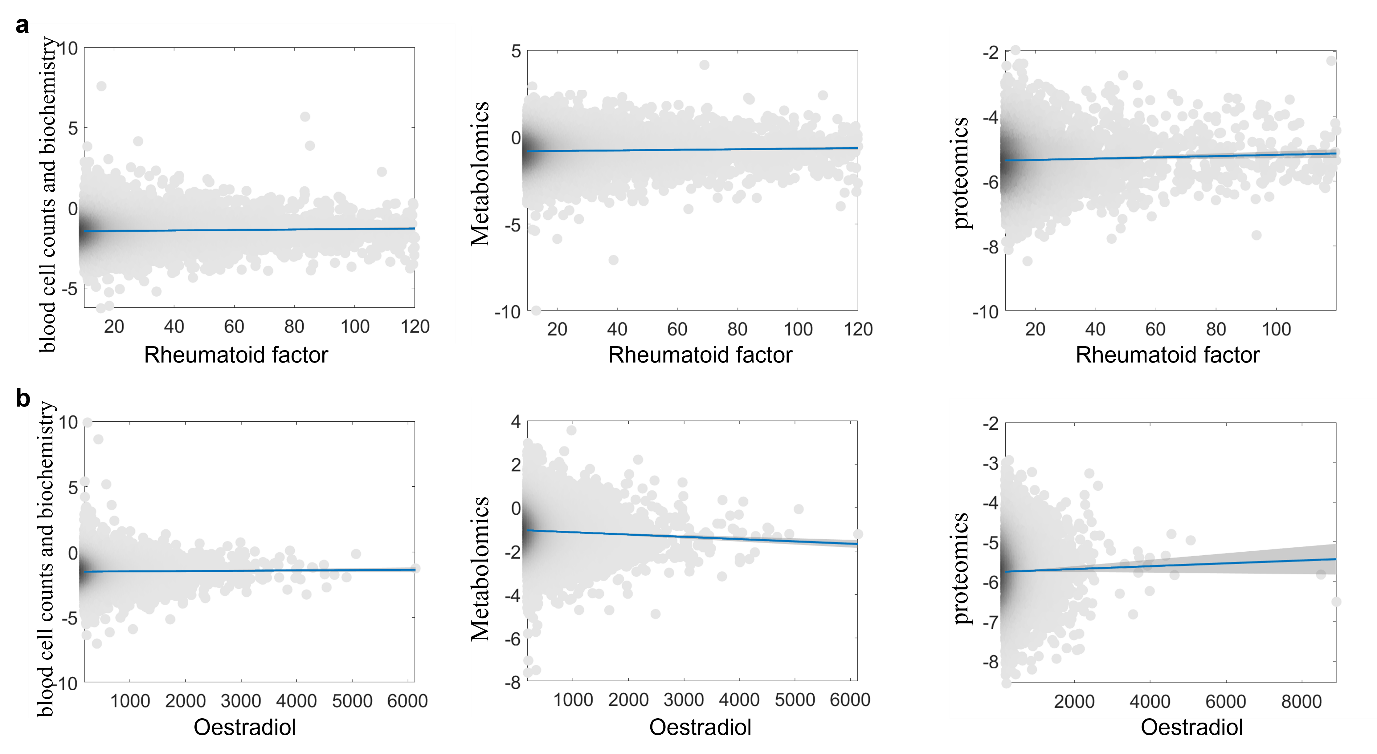
**

***Supplementary Figure 6*.** **Rheumatoid factor and oestradiol analyses.** **a,** The rheumatoid factor showed a positive correlation with blood cell counts and biochemistry (t = 3.38, p = 7.27 × 10⁻⁴), metabolomics (t = 3.63, p = 2.83 × 10⁻⁴) and proteomics (t = 2.80, p = 5.20 × 10^-3^). **b,** Oestradiol was negatively correlated with metabolomics (t = -6.01, p = 1.89 × 10⁻⁹), but showed no significant correlation with blood cell counts and biochemistry (p > 0.05) or proteomics (p > 0.05).

**
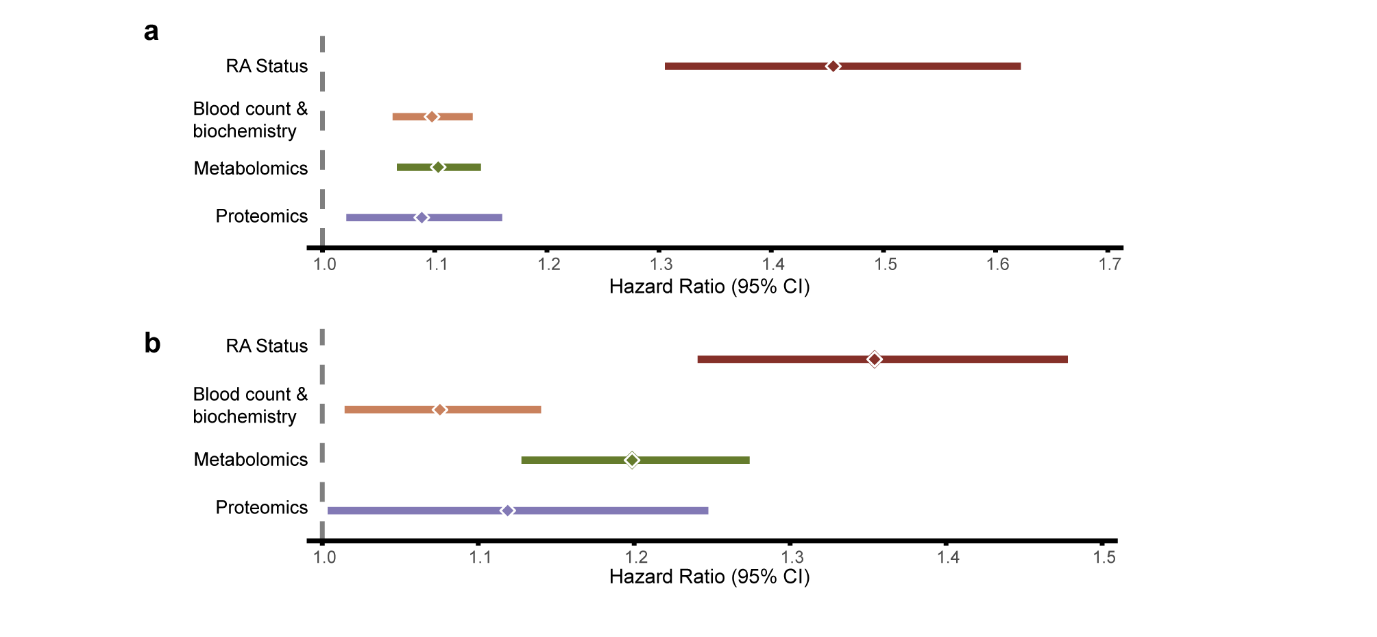
**

***Supplementary Figure 7.* Prospective and causal associations between identified multi-omic signatures and incident depression or RA after exclusion of cases occurring within two years of baseline. a,** Baseline RA status and the identified multi-omic signatures were associated with an increased risk of subsequent depression. **b,** Baseline depression status and the identified multi-omic signatures were associated with an increased risk of subsequent RA.


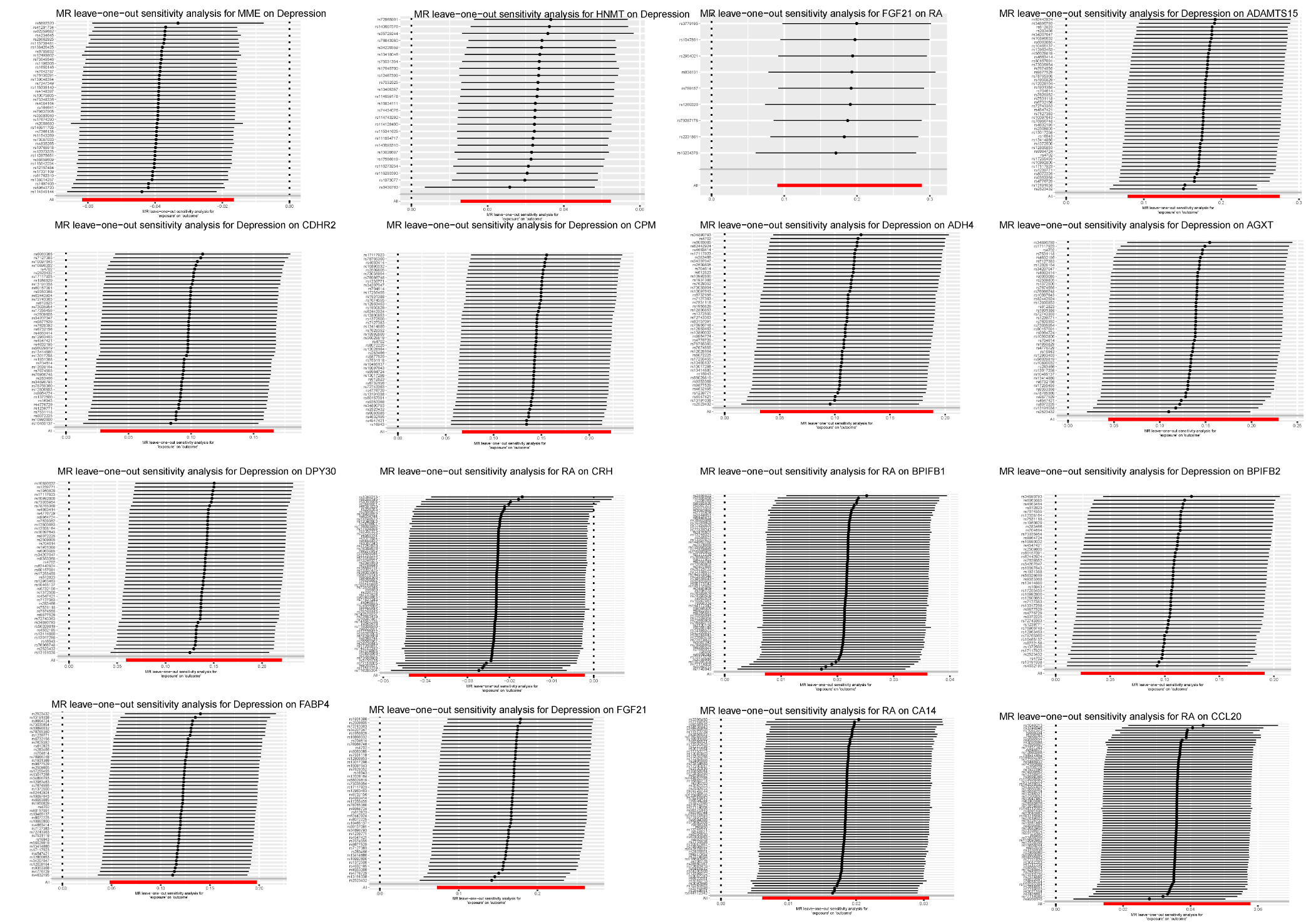


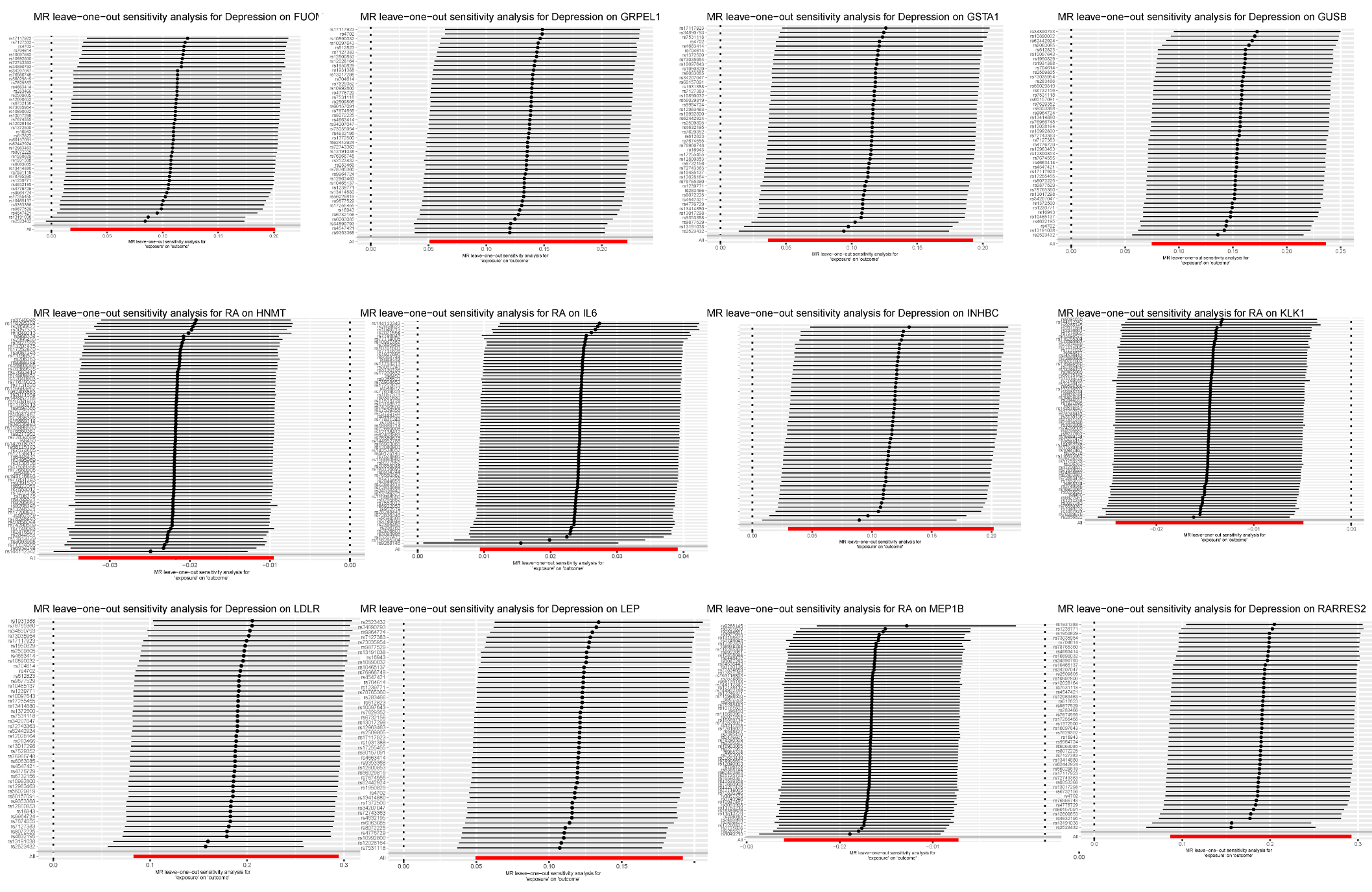


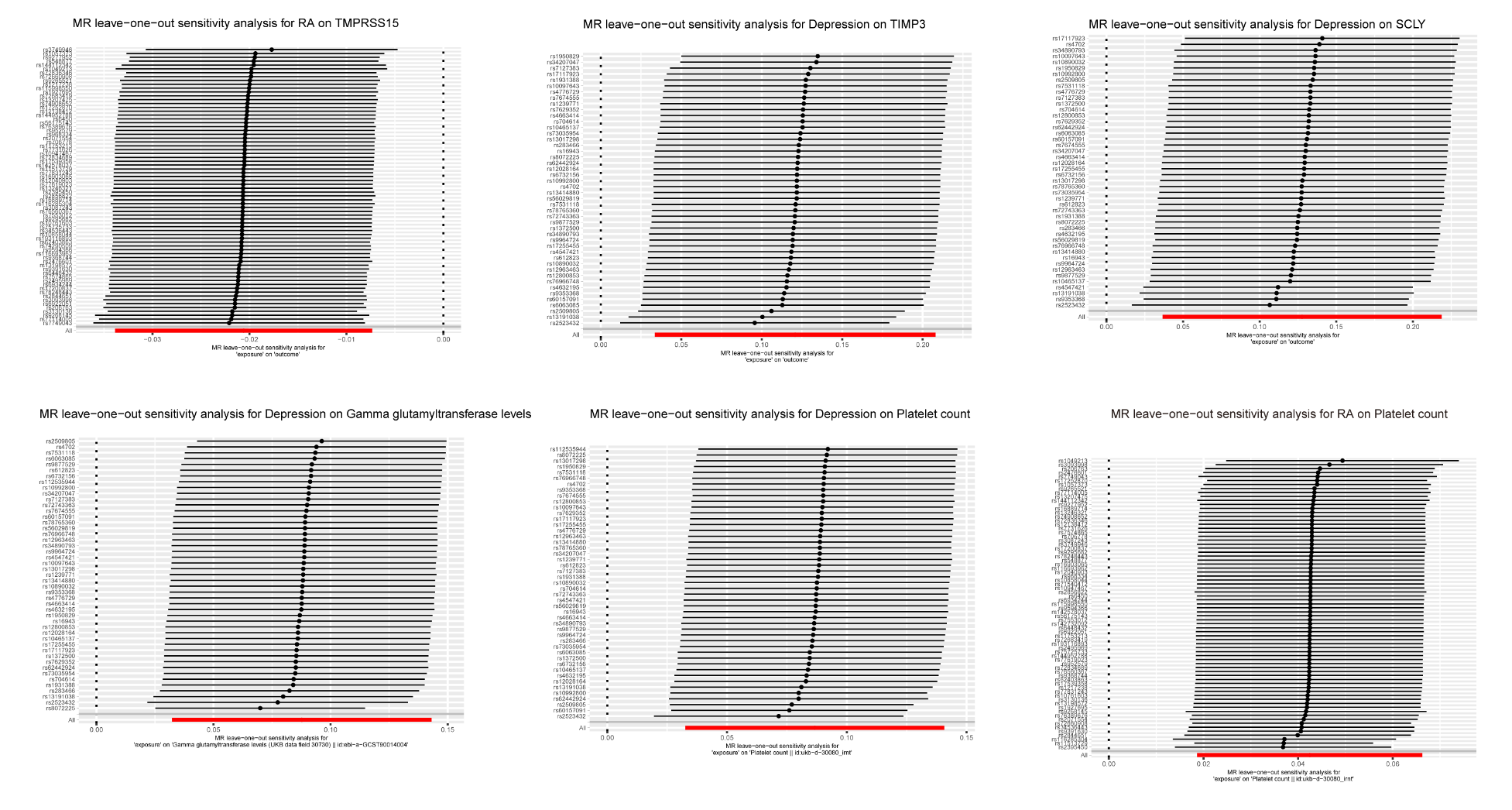


***Supplementary Figure 8.* Leave-one-SNP-out analyses.** Leave-one-SNP-out analyses of the 34 identified significant causal associations confirmed that no single SNP disproportionately influenced the overall results.

*
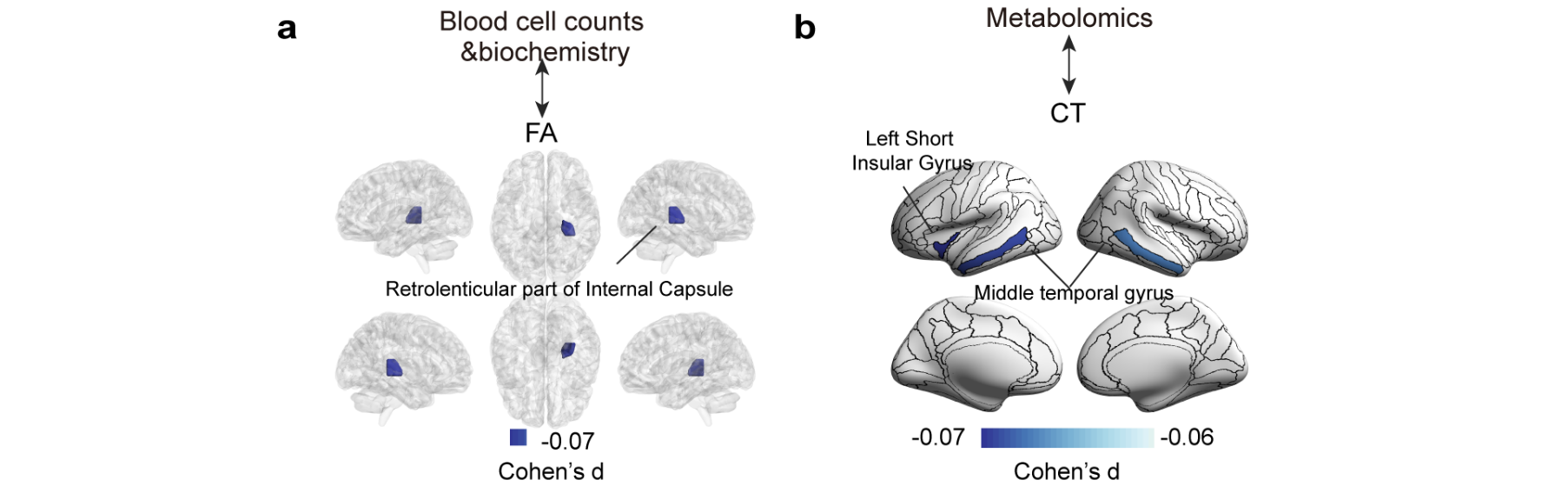
*

***Supplementary Figure 9.* Additional analyses: associations between the identified multi-omic signatures and brain structure.** blood cell count and biochemistry signatures also showed negative associations with fractional anisotropy (FA) in the left retrolenticular part of the internal capsule (*P* < 0.001, d =–0.07; Fig. 9a; Supplementary Table 5). Metabolomic signatures also showed negative associations with cortical thickness in the left short insular gyrus and bilateral middle temporal gyri (*P* < 0.001, |d| = 0.06–0.07; Fig. 9b; Supplementary Table 5).

***
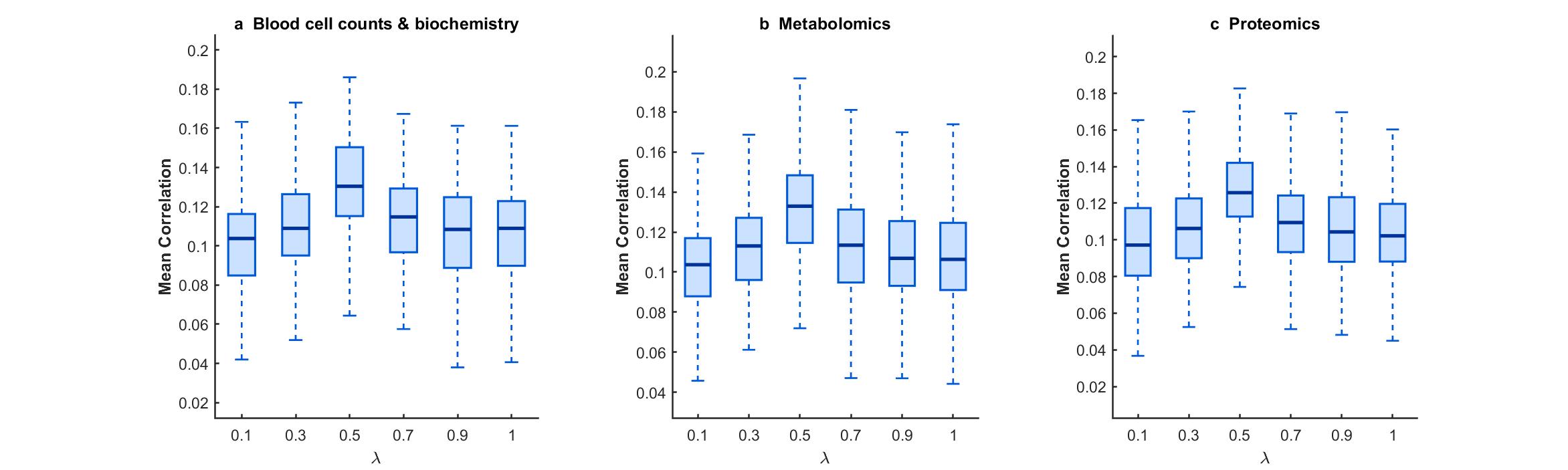
***

***Supplementary Figure 10.* Parameter tuning of the regularization parameter λ in MCCAR based on repeated cross-validation.** The correlation between the identified target independent component (IC) and MCP scores is shown across different values of λ for each modality. A repeated five-fold cross-validation framework (50 repetitions) was used, yielding 250 out-of-sample correlation estimates for each λ. Boxplots illustrate the distribution of correlation coefficients across all cross-validation iterations, with the central line indicating the median and the box representing the interquartile range. Across all modalities, λ = 0.5 yields the highest mean correlation, supporting its selection as the optimal regularization parameter.


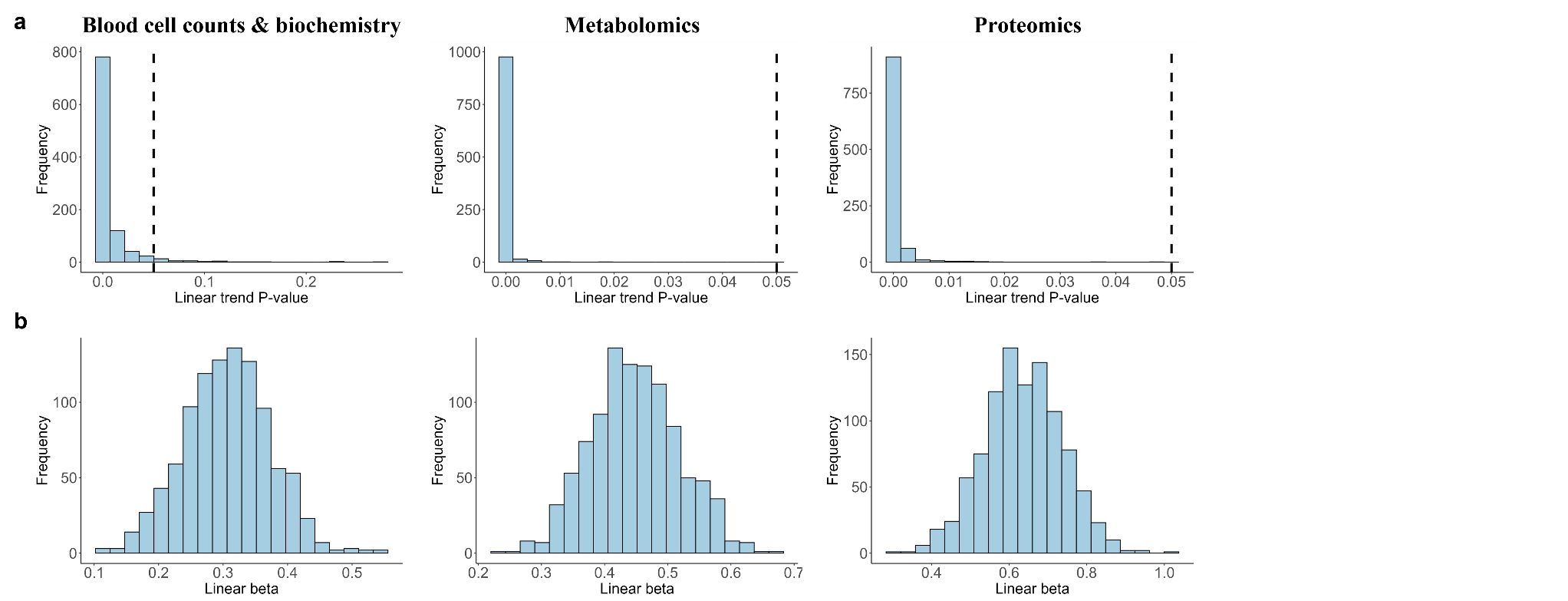


***Supplementary Figure 11.* Robustness of linear gradient to group size imbalance across omic layers.** a, Distribution of P-values for the linear trend across 1000 resampling iterations with matched group sizes, where the healthy, depression, and RA groups were resampled to the size of the comorbidity group. The dashed vertical line indicates the significance threshold (*P* = 0.05). Across omic layers, P-values were predominantly concentrated below the significance threshold, with the linear trend remaining significant in 96.4% of resampling iterations in the blood biochemistry layer and in all iterations (100%) in both the metabolomic and proteomic layers. b, Distribution of corresponding linear trend effect sizes (beta coefficients). Effect sizes were consistently in the same direction, indicating that the observed graded pattern is robust to group size imbalance.
